# L-asparaginase by filamentous fungi and yeasts from lichens and soils across maritime Antarctic islands

**DOI:** 10.1007/s00792-026-01437-2

**Published:** 2026-08-01

**Authors:** Averlane Vieira da Silva, Bárbara Emanuelle Fernandes Ferro, Vanessa Daiany Vieira Medeiros, Valdilene Canazart dos Santos, Maria Nicolle Pereira da Silva, Cledna Kaline dos Santos Duarte, Aline Cavalcanti de Queiroz, Magna Suzana Alexandre Moreira, Luiz Henrique Rosa, Alysson Wagner Fernandes Duarte

**Affiliations:** 1 Universidade Federal de Alagoas, Campus Arapiraca, Arapiraca, 57309-005 AL Brazil; 2 Universidade Federal de Alagoas, Campus A.C Simões, Maceió, 57072-970 AL Brazil; 3 Universidade Federal de Minas Gerais, Belo Horizonte, 31270-901 MG Brazil

**Keywords:** Acute Lymphoblastic Leukemia, Antarctica, Asparaginase, Bioprospecting, Polar microbiology

## Abstract

This study aimed to evaluate L-asparaginase (L-ASNase) production by fungi isolated from soils across different Antarctic islands. Out of 138 isolates, 33 filamentous fungi and 5 yeasts exhibited enzymatic activity in solid culture media. Filamentous fungi were identified by sequencing the Internal Transcribed Spacer (ITS) region, while yeasts were identified using the D1/D2 domains of the 26 S rDNA gene. The blue filamentous fungus *Antarctomyces* F13.CR.LASP exhibited a maximum L-ASNase productivity of 0.76 U/L. Regarding the yeasts, the isolate *Vishniacozyma victoriae* G.L11 reached a productivity of 0.17 U/L, while corresponding values for glutaminase and urease were 0.11 and 0.06 U/L, respectively. Furthermore, *V. victoriae* G.L11 was capable of producing the enzyme using different low-cost substrates, particularly pineapple peel waste, which yielded 5.17 U/L. In this scenario, future optimization could further enhance L-asparaginase production levels by these fungi, providing valuable insights into the biotechnological potential of Antarctic fungi.

**Graphical abstract:**

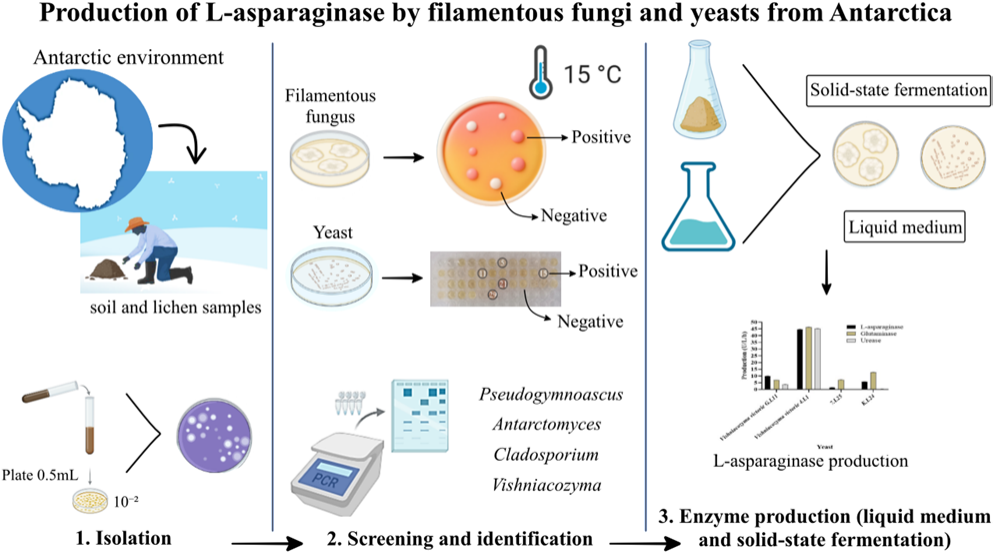

**Supplementary Information:**

The online version contains supplementary material available at 10.1007/s00792-026-01437-2.

## Introduction

Antarctic fungal communities are of significant interest to the global scientific community as they are subjected to long periods of isolation and low levels of anthropogenic disturbances. These environments safeguard a largely inaccessible biological heritage that is a prime target for unique biotechnological applications (Cavalcante et al. 2023; Silva et al. 2025), such as the search for enzymes of therapeutic interest (Ashok et al. 2019; Lima et al. 2022; Camacho et al. 2024; da Silva et al. 2026). In this context, the sustainable bioprospecting of fungal biological diversity can yield substantial benefits for humanity, including advances in disease treatment, the development of new products and processes, and food improvement (Ashok et al. 2019; Jana et al. 2025).

The rising demand for new chemotherapeutic agents with reduced allergenic effects highlights the urgent need to discover biologically active molecules to improve human health and quality of life, a task that remains one of humanity’s most significant challenges (Mapook et al. 2022). Recent investigations have positioned eukaryotic microorganisms as key sources for the discovery of therapeutic compounds. Specifically, the enzyme L-asparaginase is a crucial biopharmaceutical used in the treatment of Acute Lymphoblastic Leukemia (ALL), and its production by Antarctic fungi has been increasingly studied in recent years (Lima et al. 2022; Andrade et al. 2023). While L-asparaginase can be obtained from various natural sources, including plants and animals, microbial sources are considered the most efficient and reliable due to their diversity, ease of manipulation, and the feasibility of optimizing culture conditions for production (Seleena et al. 2023).

In this regard, the search for eukaryotic microorganisms is essential, and fungi are particularly attractive due to the standardization of efficient, large-scale cultivation processes within short timeframes. Furthermore, as eukaryotes, they possess various post-translational modifications that make them a significant resource for L-asparaginase production (Sarquis et al. 2004; Andrade et al. 2023). Some studies have documented the production of glutaminase- and urease-free L-asparaginases by filamentous fungi and yeasts (Ashok et al. 2019; Andrade et al. 2023; Sánchez-Moguel et al. 2023). However, given the vast microbial richness of the Antarctic continent, such findings can still be potentially expanded. The present study prospected L-asparaginase production by fungal isolates from the Antarctic ecosystem, aiming to identify eukaryotic organisms capable of producing the enzyme with minimal glutaminase and urease activity.

## Materials and methods

### Collection of biological material

The study utilized filamentous fungi and yeasts isolated from various environmental samples from Antarctica. Filamentous fungi were isolated from soil samples collected on Deception and King George Islands, within the South *Shetland* Archipelago, during two expeditions of the Brazilian Antarctic Program (PROANTAR) in 2017 and 2019 (Table 1). Soils were obtained from meltwater regions and phosphatic (Supplementary Fig. 1). Samples were placed in sterile collection bags and maintained under freezing conditions until the processing and microbial isolation stages at the Laboratory of Microbiology, Immunology, and Parasitology (LabMip) – UFAL, Arapiraca campus.

The yeasts used in this study were isolated from lichen samples in previous studies and molecularly identified by Silva et al. (2022a, b). These isolates are preserved at –80.0 °C and were retrieved starting from the screening stage for this research.

**Table 1:** Collection sites of soil samples on Deception and King George Islands, Maritime Antarctica

Substrate	Local	GPS data	Collection date
Thawed ground	Crater Lake	Deception Island	12/2017
	Alargado	Deception Island	
	Fumarole Bay	Deception Island	
Phosphate soil	Yellow Point: Dog house	King George Island	12/2019
	Yellow Point: Morro da Cruz	King George Island	
	Yellow Point: Punta Plaza	King George Island	

### Isolation of filamentous fungi using differential selective media for L-asparaginase

The isolation of filamentous fungi was performed by homogenizing 10.0 g of soil in 90 mL of sterile 0.9% saline solution. The suspension was then placed in an orbital shaker at 125 rpm (revolutions per minute) for 30.0 min at 15.0 °C. A 500 µL aliquot, with serial dilutions until a concentration of 10⁻² was reached, was inoculated onto Petri dishes containing a selective differential medium for the isolation of L-asparaginase-producing fungi. The Modified Czapek Dox (MCD) medium, supplemented with a pH indicator (0.009% phenol red), was composed of (g/L): glucose 2.0, L-asparagine 10.0, KH_2_PO_4_ 1.52 – KCl 0.52 – MgSO_4_.7H_2_O 0.52, and trace amounts of FeSO_4_.7H_2_O, ZnSO_4_.7H_2_O e CuNO_3_.3H_2_O (0.001 g/L each), 20.0 g/L agar dissolved in distilled water (Mahajan et al. 2013). The final pH of the medium was adjusted to 5.5 (Gulati et al. 1997; Doriya and Kumar 2018). Fungal growth was monitored during incubation at 15.0 °C for up to 30 days.

### Confirmation of L-asparaginase production in fungal isolates

#### Screening of yeast isolates in 96-well deep-well plates

The screening of L-asparaginase (L-ASNase) producing microorganisms is commonly performed using solid Czapek Dox (CD) medium (Mahajan et al. 2013). However, researchers have observed that the ability of certain fungi to produce L-ASNase on solid media may differ from their performance in liquid media therefore, this methodology was specifically applied to yeasts. A total of 29 Antarctic yeast isolates were selected for initial screening based on their vigorous growth under established experimental conditions. These yeasts were inoculated into 96-well deep-well microplates containing Czapek Dox broth.

The plates were sealed with silicone lids and maintained under constant agitation at 125 rpm for 7 days (168 h) at 15.0 °C. Sterile culture media without yeast inoculum were used as contamination controls. On the seventh day, each isolate was qualitatively evaluated for L-ASNase production using the L-aspartyl-β-hydroxamic colorimetric method. The selection criterion was based solely on the intensity of the red color after development with ferric chloride/TCA/HCl, without quantitative spectrophotometric assessment (Freire et al. 2020).

#### Differential L-asparaginase screening

Differential L-asparaginase screening was performed on agar plates using Modified Czapek Dox (MCD) medium. For filamentous fungi, a 3.0 mm fungal disk was placed at the center of the medium. For yeasts, the inoculum was standardized using a UV/Vis spectrophotometer (Model UV-5100, Tecnal) at an absorbance of 600 nm (OD₆₀₀). A 10 µL aliquot was then inoculated at the center of the medium. The plates were incubated at 15.0 °C for up to 15 days. L-ASNase activity was assessed using the Enzymatic Index (EI), defined as the ratio between the diameter of the halo indicating enzymatic activity and the diameter of the filamentous fungal or yeast colony.

#### Confirmation of L-ASNase production in liquid-based with pH indicator

The filamentous fungi and yeasts identified as potential L-asparaginase producers in the previous steps were cultivated in Erlenmeyer flasks containing 50 mL of CD broth, supplemented with bromothymol blue as a pH indicator (pH 5.5) and proline as the sole nitrogen source, to infer L-ASNase production via pH shifts. The flasks were placed in an orbital shaker for 7 days at 15.0 °C. After this period, the supernatants were analyzed. The CD medium is initially orange and turns blue under basic conditions. Since L-ASNase production and the subsequent degradation of asparagine release ammonia thereby increasing the pH the color change to blue indicates the production of the target enzyme. Uninoculated CD media and media without a nitrogen source (proline) were used as controls (Mahajan et al. 2013).

### Quantification of L-ASNase, glutaminase, and urease using the hydroxylamine method

Enzymatic activity was quantified using the L-aspartyl-β-hydroxamic acid method, as described by Drainas et al. (1977). The reagents required for the enzymatic assay included: 20 mM Tris-HCl buffer (standardized at pH 8.6) 100 mM solutions of L-asparagine, glutamine, and urea a 1 M hydroxylamine solution (pH 7.0) and a color reagent solution composed of 5% (w/v) trichloroacetic acid TCA - C_2_HCl_3_O_2_ (50.0 g/L), 10% (w/v) ferric chloride (FeCl_3_, 100.0 g/L) in 0.66 M HCl, and β-aspartyl hydroxamic acid (AHA).

For the assay, the test reaction consisted of 0.3 mL of 20 mM Tris-HCl buffer (pH 8.6), 0.1 mL of L-asparagine solution (100 mM), 0.1 mL of hydroxylamine solution (1 M) neutralized with 3 M sodium hydroxide (NaOH), and 0.5 mL of the crude enzymatic extract. The reaction was incubated at 37.0 °C for 2 h in a water bath (El-Naggar et al. 2016), after which it was terminated by the addition of 0.25 mL of the color reagent, followed by centrifugation at 13.000 rpm for 5 min. Absorbance was measured at 500 nm using a Loccus LMR Flex UV-Vis 96-well microplate reader. Blank reactions were conducted to determine the AHA concentration. All assays were performed in triplicate (Freitas et al. 2021). A standard curve was constructed using at concentrations of 0.10, 0.25, 0.50, 0.75, 1.00, 1.50 µmol of AHA, plus 0.50 mL of the ferric chloride color solution with an R^2^ (coefficient of determination) value 0.9974 (Supplementary Fig. 2).

To quantify glutaminase and urease activities, the same methodology was followed, with substitution of the 100 mM L-asparagine solution with glutamine and urea at the same concentration, respectively. Results are presented as the mean ± standard deviation of enzymatic activity. One unit (U) of L-asparaginase was defined as the amount of enzyme required to form 1 µmol of L-aspartyl-β-hydroxamate per liter per minute at 37.0 °C. L-asparaginase production can be expressed in different units; however, units per culture volume (U/mL or U/L), units per cell dry mass (U/g or U.mg), or even enzyme units per mg of total protein (U/mg) have been adopted. Following this reasoning, the unit used in this research was U/L (culture volume), the same standard used by Freire et al. (2020).

Total protein content was quantified using the BCA Protein Assay kit (Pierce™ BCA) colorimetric method. Briefly, 200 µL of the freshly prepared working reagent was added to a 25 µL aliquot of the crude enzyme supernatant in triplicate in a 96-well microplate. After incubation at 37.0 °C for 30 min, the absorbance (purple color) was measured at 562 nm. A bovine serum albumin (BSA) standard curve (0–2.0 mg/mL) was constructed in triplicate to determine the total protein concentration in the samples.

### Evaluation of L-asparaginase production using different low-cost substrates

The best L-asparaginase-producing isolate from the liquid media assays was evaluated for its enzymatic production capacity using different low-cost substrates. Five agro-industrial residues were analyzed: wheat bran, rice flour, sugarcane bagasse, corn straw and pineapple peel. The peels and bagasse were washed, dried in an oven at 60.0 °C, and subsequently ground and sieved to achieve a particle size of approximately 0.5–2 mm. The efficacy of these agro-industrial residues as substrates for L-asparaginase production was then assessed.

Solid-state fermentation (SSF) was carried out in 125 mL Erlenmeyer flasks containing 5.0 g of each dry substrate. The flasks were sterilized and subsequently supplemented with trace salts FeSO_4_.7H_2_O, ZnSO_4_.7H_2_O and Cu(NO₃)₂·3 H₂O – 0.001 g/L) to reach a target moisture content of 70%, accounting for the inoculum volume in the final liquid addition. Thus, 10.67 mL of the trace salt solution and 1 mL of the yeast suspension were added (total volume of 11.67 mL). The yeast inoculum was standardized using a UV-Vis spectrophotometer at an absorbance of 600 nm (OD₆₀₀). Finally, the flasks were incubated under stationary conditions at 15.0 °C for 7 days. Enzyme production was expressed in units per gram of dry substrate (U/gds).

The humidity (%) was assessed by weighing 5.0 g of each dried substrate in a porcelain crucible before and after being subjected to a temperature of 105.0 °C for 6 h.

The moisture content of the samples was calculated according to the Eq. 1:

1 $$\:\frac{\left(starting\:weight-final\:sample\:weight\right)*100}{starting\:weight}$$

The apparent density of 5.0 g of each sample was placed in a 100 mL test tube to evaluate the density the material occupies without compaction. The results were obtained from Eq. 2:

2 $$\:\frac{mass\left(g\right)}{occupied\:volume}$$

(Orzua et al. 2009; Agarwal et al. 2023).

### Principal Component Analysis (PCA) for evaluating solid-state fermentation substrates

PCA was performed by selecting the two most significant principal components (PC1 and PC2) using the SRplot platform (https://www.bioinformatics.com.cn/srplot). PCA was employed to evaluate the relationship between the chemical composition of the substrates used in solid-state fermentation and the potential production of L-asparaginase. PC1 and PC2, which accounted for most of the total data variability, were used to construct a biplot. This allowed for the visualization of groupings among the substrates and the identification of which chemical variables contributed most to their separation (Tang et al. 2023; Šelo et al. 2024).

### Molecular identification of fungal isolates

Genomic DNA was extracted using the modified salting-out method originally proposed by Bandehpour et al. (2023). For filamentous fungi, the internal transcribed spacer (ITS) region was amplified by Polymerase Chain Reaction (PCR) following the methodology of White et al. (1990), using the universal primers ITS-1 (5’-TCC GTA GGT GAA CCT GCG G-3’) and ITS-4 (5’-TCC TCC GCT TAT TGA TAT GC-3’). For yeasts, the D1/D2 domains of the 26 S rRNA gene (600 bp) were amplified using primers NL-1 (5’-GCA TAT CAA TAA GCG GAG GAA AAG-3’) and NL-4 (5’-GGT CCG TGT TTC AAG ACG G-3’) (Fell et al. 2000). The final PCR volume was 25 µL, containing: 1–5 µL of DNA (10–100 ng), 0.5 µL of each primer, 2.5 µL of PCR buffer, 1.25 µL of 50 mM MgCl_2_ 0.4 µL of 1.25 mM dNTPs, and 0.4 µL of Taq DNA polymerase (5 U), with the final volume adjusted with DNA-free sterile water. PCR reactions were performed in a thermal cycler using specific conditions for each genomic region and fungal group. Amplicons were analyzed by 1.0% agarose gel electrophoresis in 1.0x TBE buffer at 120 V for 50 min, followed by staining with SYBR Green solution. Gels were visualized under ultraviolet light and photodocumented. Sequencing was performed using the Sanger method.

The resulting sequences were evaluated for quality and manually edited using Chromas (version 2.6.6) available at: technelysium.com.au/wp/chromas-version-history. Subsequently, sequences were compared with those deposited in GenBank and international culture collections using the BLASTn algorithm (NCBI), available at: blast.ncbi.nlm.nih.gov/Blast.cgi. For phylogenetic tree construction, sequences were aligned using BioEdit (version 7.2) available at: bioedit.software.informer.com/7.2/, and evolutionary distances were evaluated with MEGA X (Kumar et al. 2018), available at https://www.megasoftware.net/downloads/dload_win_gui. The Neighbor-Joining algorithm was applied, and the method was used to estimate evolutionary distances between L-asparaginase isolates (Saitou and Nei 1987). Bootstrap analysis was performed with 1.000 replicates. Information regarding fungal taxonomic hierarchies was obtained from the NCBI and MycoBank databases, (ncbi.nlm.nih.gov/genome/browse#!/prokaryotes/), MycoBank (www.mycobank.org/).

### Statistical analysis

Analysis of variance (ANOVA) was performed using a single approach to compare the differences between the means of an independent variable between the low-cost substrates using the Scott-Knott’s test. All significant differences between the arithmetic means were determined by the 5% probability test using the SISVAR 5.8 statistical software (Scott and Knott 1974).

## Results

### Fungal isolation

**Fig. 1:**
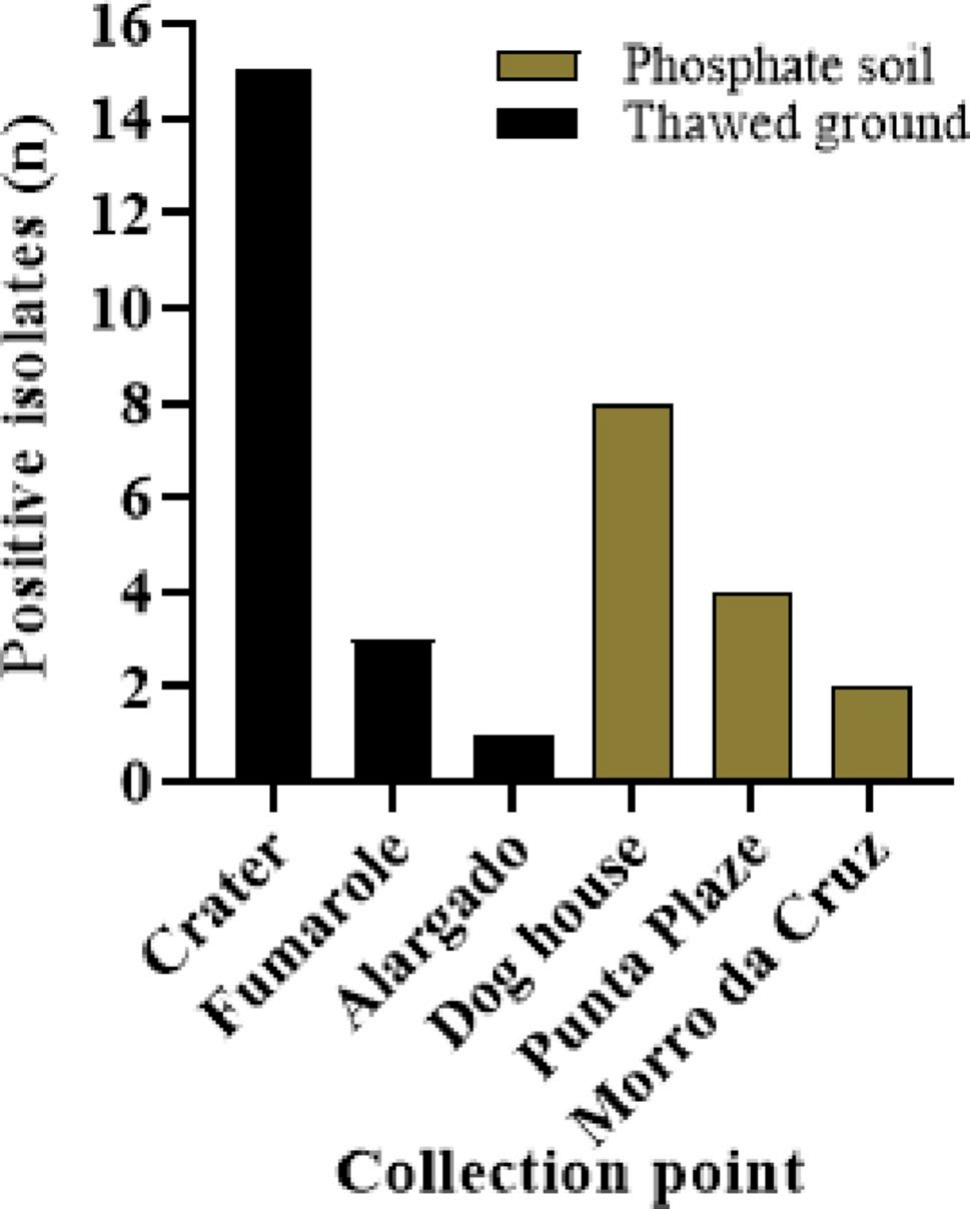
Number of fungal isolates positive for L-asparaginase obtained from selective isolation of Antarctic soils

A total of 138 filamentous fungi were isolated from various Antarctic soil samples (Fig. 1), of which 33 were selected for the subsequent stage (plate screening). The flowchart of the total number of isolates in this study is presented in Supplementary Fig. 3.

### Bioprospecting of L-ASNase-producing yeasts

**Fig. 2:**
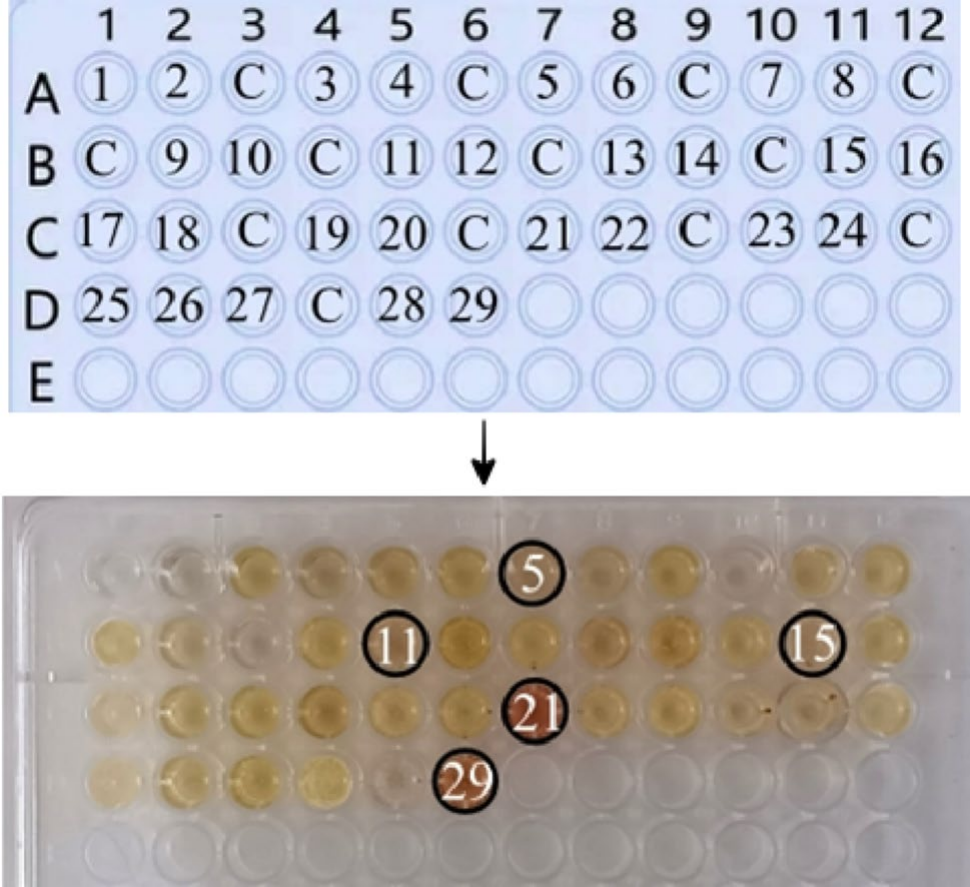
Selective screening of Antarctic L-ASNase-producing yeasts. Numbers indicate the corresponding isolates. The five producing strains are highlighted in red. The letter “C” represents control (without microorganisms)

The yeast isolates (*n* = 29) were obtained from previous studies and selected for this and subsequent research stages. The yeasts were inoculated into microplates containing Czapek Dox broth, only five isolates (17.24%) exhibited significant activity (Fig. 2). These five strains were selected for the solid medium plate assay stage.

### Qualitative screening on solid media

**Fig. 3:**
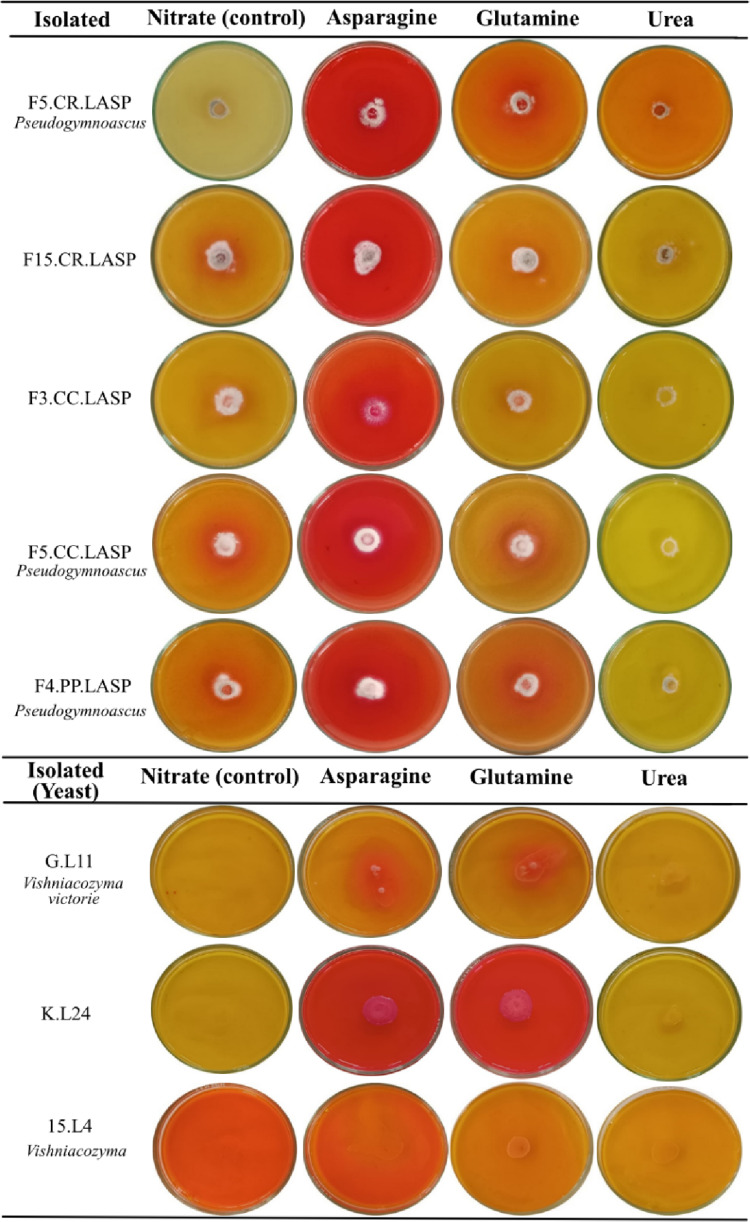
Visualization of selective screening across different nitrogen sources for filamentous fungi and yeasts

A total of 33 filamentous fungi and 5 yeasts with the highest activities were selected for evaluation across different nitrogen sources: nitrate, asparagine, glutamine, and urea (Supplementary Fig. 4). Regarding the yeasts, three isolates showed growth on asparagine and glutamine (Fig. 3). The standout isolates in this stage were the filamentous fungi *Pseudogymnoascus* F5.CR.LASP, F15.CR.LASP (unidentified), F3.CC.LASP (unidentified), *Pseudogymnoascus* F5.CC.LASP, *Pseudogymnoascus* F4.PP.LASP, and the yeast *Vishniacozyma* l5.L4 with activity of 5.45; 4.10; 2.67; 4.74; 4.49 and 4.48, respectively (Table 2). These isolates exhibited an Enzymatic Index (EI) for L-asparaginase while being urease-free and showing little to no glutaminase.

**Table 2:** Enzymatic Index (EI) of L-ASNase, glutaminase, and urease for filamentous fungi and yeasts from Antarctica

Collection point	Isolated	Enzymatic Index (IE)			
	Filamentous fungi	Nitrate	Asparagine	Glutamine	Urea
Deception: Crater Lake	**F1.CR.LASP**	–	4.17	3.70	–
	**F2.CR.LASP**	–	2.73	1.73	–
	**F3.CR.LASP**	–	1.43	1.02	–
	**F4.CR.LASP**	1.93	5.88	5.26	–
	**F5.CR.LASP**	–	5.45	1.96	–
	**F6.CR.LASP**	2.47	5.26	5.45	–
	**F7.CR.LASP**	1.59	4.44	4.41	–
	**F8.CR.LASP**	–	4.84	4.84	–
	**F9.CR.LASP**	2.04	5.88	5.27	–
	**F10.CR.LASP**	1.98	5.09	3.73	–
	**F11.CR.LASP**	–	5.55	2.64	–
	**F12.CR.LASP**	2.00	4.31	3.11	–
	**F13.CR.LASP**	–	1.95	1.34	–
	**F14.CR.LASP**	1.31	3.79	2.64	–
	**F15.CR.LASP**	1.09	4.10	1.18	–
Yellow Point: Dog house	**F1.CC.LASP**	–	5.66	3.58	–
	**F2.CC.LASP**	1.53	6.00	5.38	–
	**F3.CC.LASP**	1.32	2.67	–	–
	**F4.CC.LASP**	1.81	4.52	4.15	–
	**F5.CC.LASP**	2.20	4.74	1.81	–
	**F6.CC.LASP**	1.90	4.38	2.35	–
	**F7.CC.LASP**	2.14	5.76	4.34	–
	**F8.CC.LASP**	1.01	4.47	2.85	–
Deception: Alargado	**F1.AL.LASP**	–	3.61	1.42	–
Yellow Point: Punta Plaza	**F1.PP.LASP**	2.29	5.88	5.56	–
	**F2.PP.LASP**	–	4.10	3.73	–
	**F3.PP.LASP**	1.50	5.62	3.11	–
	**F4.PP.LASP**	1.74	4.49	1.75	
Yellow Point: Abaixo Morro da Cruz	**F1.ADC.LASP**	2.28	4.07	5.37	–
	**F2.ADC.LASP**	2.45	5.66	4.48	–
Deception: Fumarole Bay	**F1.FBP1.LASP**	1.69	4.24	1.79	–
	**F1.FBP2.LASP**	1.72	4.47	5.55	–
	**F2.FBP2.LASP**	2.14	4.07	3.41	–
	**Yeast**				
Greenwich Island	**7.L25**	–	–	–	–
Deception Island	**G.L11**	–	1.26	1.30	–
–	**K.L24**	–	3.49	4.28	–
King George Island	**4.L1**	–	–	1.27	–
Esperanza Base	**15.L4**	5.17	4.84	–	–

### Taxonomic identification

**Fig. 4:**
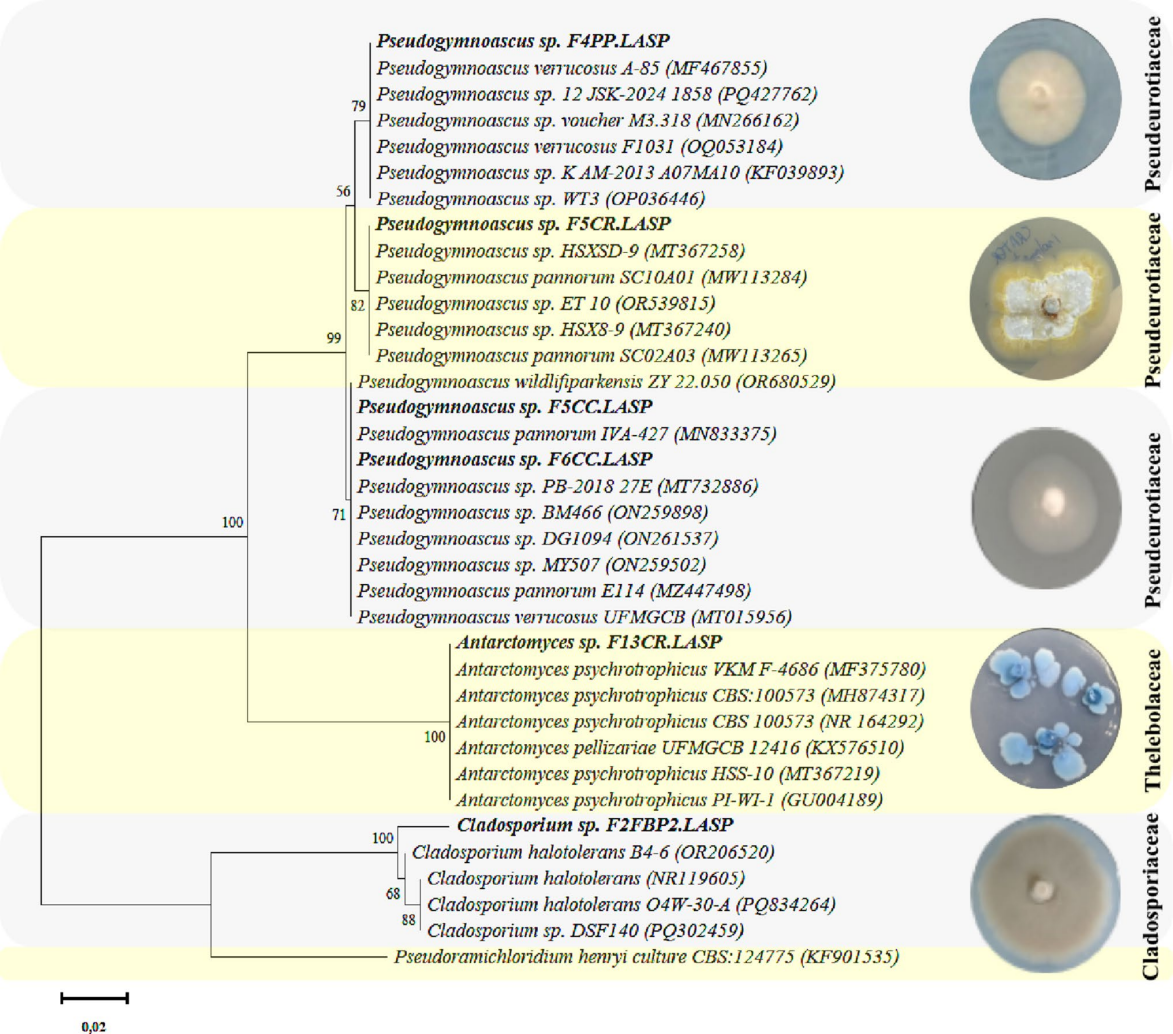
Phylogenetic tree constructed using the Neighbor-Joining method for filamentous fungi isolates from soils of different locations across the *South Shetland*. Isolates are highlighted in bold, along with their respective colonies and families. Bootstrap values above 50% are shown

Molecular identification revealed that the primary L-asparaginase-producing fungi belong to the distinct genera *Pseudogymnoascus*, *Antarctomyces*, and *Cladosporium* (Fig. 4). Notably, the ITS sequences of these fungi recovered from soil samples exhibited BLASTn identities, confirming that they belong to the phylum Ascomycota, spanning three different genera. Sequences were deposited into GenBank database with a unique accession number: PZ437408-PZ437413. These genera are potentially pioneer L-asparaginase producers, making these results promising. The genera identified in this study have been described by other authors for their potential as biosurfactant producers, particularly the endemic species *Antarctomyces psychrotrophicus* (Silva et al. 2024).

Regarding the yeasts, the top-performing isolates were identified as *Vishniacozyma victoriae* 4.L1 (isolated from the lichen *Usnea aurantiacoatra* collected on King George Island), *Vishniacozyma victoriae* G.L11 (isolated from the lichen *Usnea capillacea* collected on Deception Island), and *Vishniacozyma* l5.L4 (isolated from the lichen *Polycauliona candelaria* collected on the Antarctic Peninsula). These were identified in previous studies (Silva et al. 2022a, b), as shown in Table 3.

### Taxonomic identification and prevalance

Molecular identification based on partial sequencing of the ITS rRNA gene showed a prevalence of the genus *Pseudogymnoascus* (*n* = 4). The fungi obtained from Antarctic soils, identified through BLASTn sequence comparison and GenBank database matching, are presented in Table 3.

**Table 3:** Taxonomic affiliation of L-asparaginase-producing Antarctic filamentous fungi

Identification: filamentous fungi (GenBank)	Closest species blast (Genbank)	(pb)	Cover (%)	Similarity (%)
*Pseudogymnoascus* sp. F4.PP.LASP (PZ437408)	*Pseudogymnoascus verrucosus* A-85 (MF467855)	433	100	100 (433/433)
*Cladosporium* sp. F2.FBP2.LASP (PZ437410)	*Cladosporium halotolerans* B4-6 (OR206520)	301	100	98.01 (295/301)
*Antarctomyces* sp. F13.CR.LASP (PZ437411)	*Antarctomyces psychrotrophicus* VKM F-4686 (MF375780)	614	100	99.51 (612/615)
*Pseudogymnoascus* sp. F5.CR.LASP (PZ437413)	*Pseudogymnoascus* sp. HSXSD-9 (MT367258)	328	100	99.70 (327/328)
*Pseudogymnoascus* sp. F5.CC.LASP (PZ437409)	*Pseudogymnoascus pannorum* IVA-427 (MN833375)	430	100	100 (430/430)
*Pseudogymnoascus* sp. F6.CC.LASP (PZ437412)	*Pseudogymnoascus* sp. PB-2018 (MT732886)	235	100	100 (235/235)

### Qualitative screening for confirmation of L-ASNase production in liquid-based

**Fig. 5:**
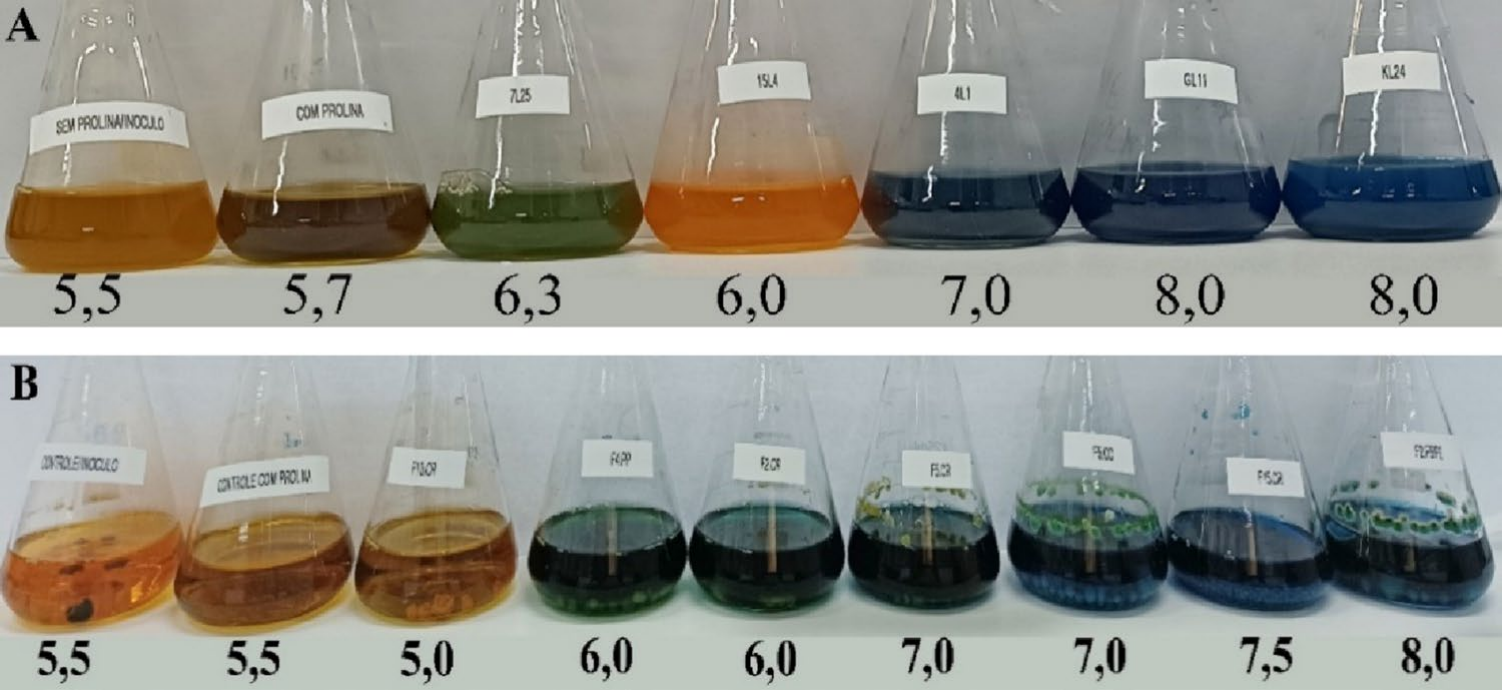
Selective screening of Antarctic yeasts (**A**) and filamentous fungi (**B**) in liquid-based, and their respective pH values

The color shift in the liquid culture medium was evident, turning blue after 7 days of incubation in cases of enzymatic production. Additionally, variations in the pH of the supernatant from the Antarctic microorganisms were observed. These results confirmed the findings from previous screenings. The yeasts *Vishniacozyma victoriae* 4.L1, *Vishniacozyma victoriae* G.L11, and K.L24 (unidentified) exhibited the highest color intensity and elevated pH values of 7.0, 8.0, and 8.0, respectively (Fig. 5A), while the control samples showed little to no increase, with final pH values of 5.5 and 5.7. Similarly, the filamentous fungi exhibited changes in coloration and an increase in medium pH to 7.0, 7.5, and 8.0, indicating enzymatic production for isolates F5.CR.LASP, F5.CC.LASP, F15.CR.LASP, and F2.FBP2.LASP, respectively, compared to the control sample with a final pH of 5.5 (Fig. 5B).

### Quantitative evaluation of L-ASNase, glutaminase, and urease

**Fig. 6:**
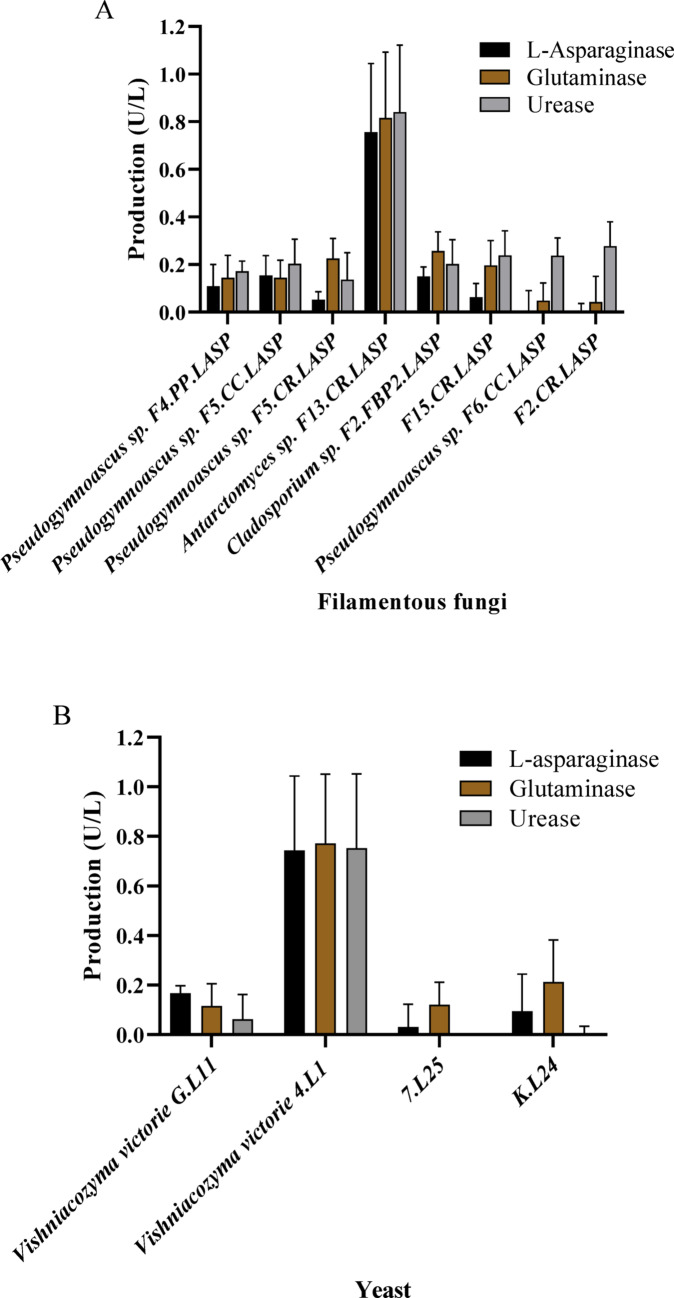
Enzymatic activity of L-asparaginase, glutaminase and urease in filamentous fungi (**A**) and yeasts (**B**)

The top-performing filamentous fungi (*n* = 8) and yeasts (*n* = 4) exhibited varying levels of target enzymatic production in liquid media. The filamentous fungus *Antarctomyces* F13.CR.LASP reached a maximum L-ASNase value of 0.76 U/L, however, it also showed high levels of glutaminase and urease, with 0.82 and 0.84 U/L, respectively (Fig. 6A). Regarding the yeasts, the isolate *Vishniacozyma victoriae* G.L11 stood out, with an L-ASNase productivity of 0.17 U/L, while glutaminase and urease values were 0.11 and only 0.06 U/L, respectively (Fig. 6B). Enzymatic activity production, along with their respective total protein values can be found in Supplementary Tables 1 and 2.

### L-asparaginase production using low-cost substrates

**Fig. 7:**
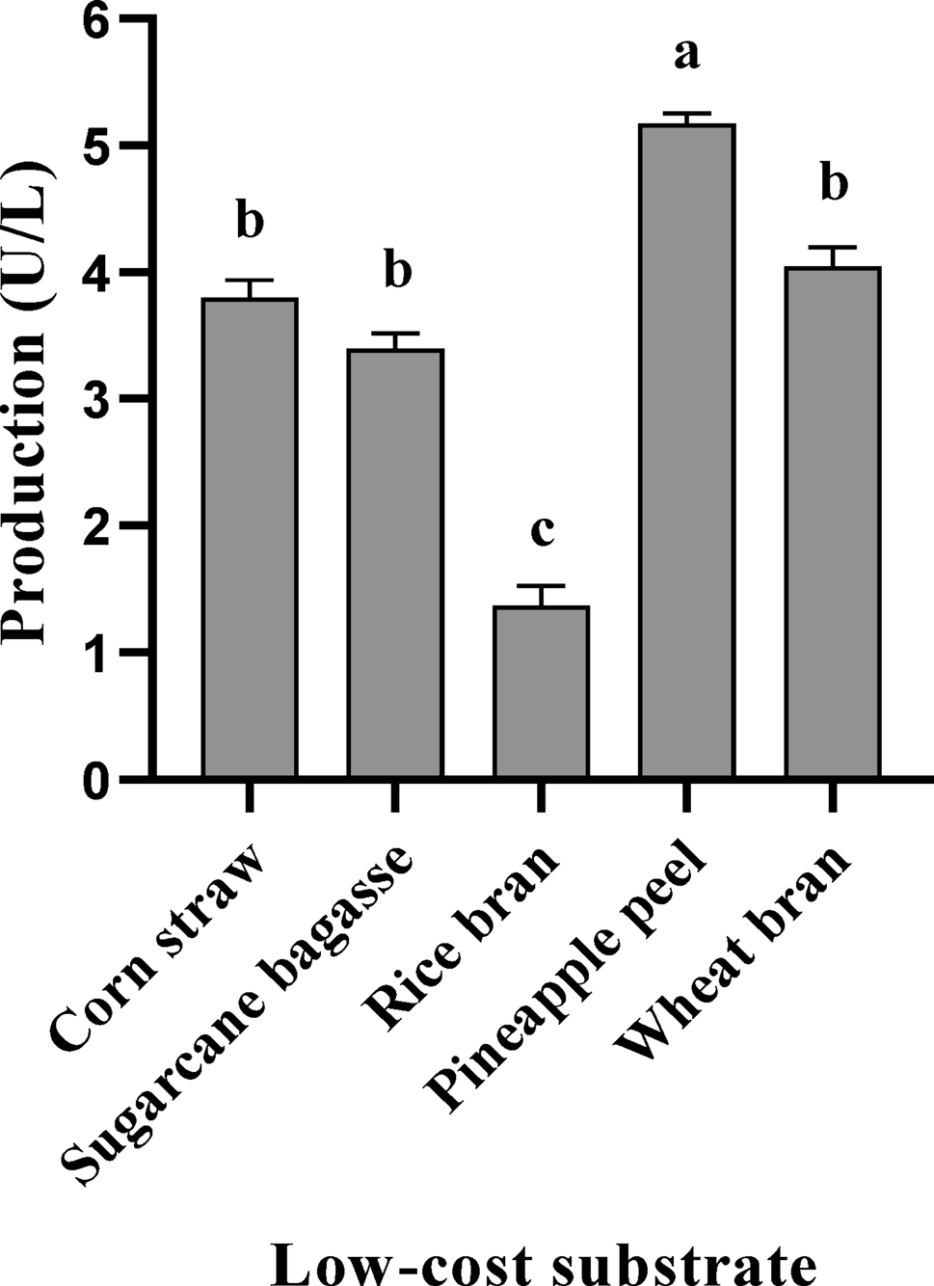
L-asparaginase production by *Vishniacozyma victoriae* G.L11 using different low-cost substrates. *Means followed by equal letters in the columns belong to the same grouping by Scott-Knott’s test, at 5% probability. Pineapple peel (U/gds = 0.05171), wheat bran (U/gds = 0.04048), corn straw (0.03798), sugarcane bagasse (U/gds = 0.03397), rice flour (U/gds = 0.01367)

L-asparaginase produced by *Vishniacozyma victoriae* G.L11 was observed on several low-cost substrates. Pineapple peel was the most effective inducing substrate, resulting in a maximum production of 5.17 U/L and consequently differing statistically from the others. On the other hand, the substrates corn straw, sugarcane bagasse and wheat bran did not show a statistically significant difference between themselves. In contrast, rice flour resulted in the lowest production, of 1.36 U/L (Fig. 7, Supplementary Table 3). The ANOVA summary can be accessed in Table 3 of the supplementary material.

### Principal component analysis (PCA)

**Fig. 8:**
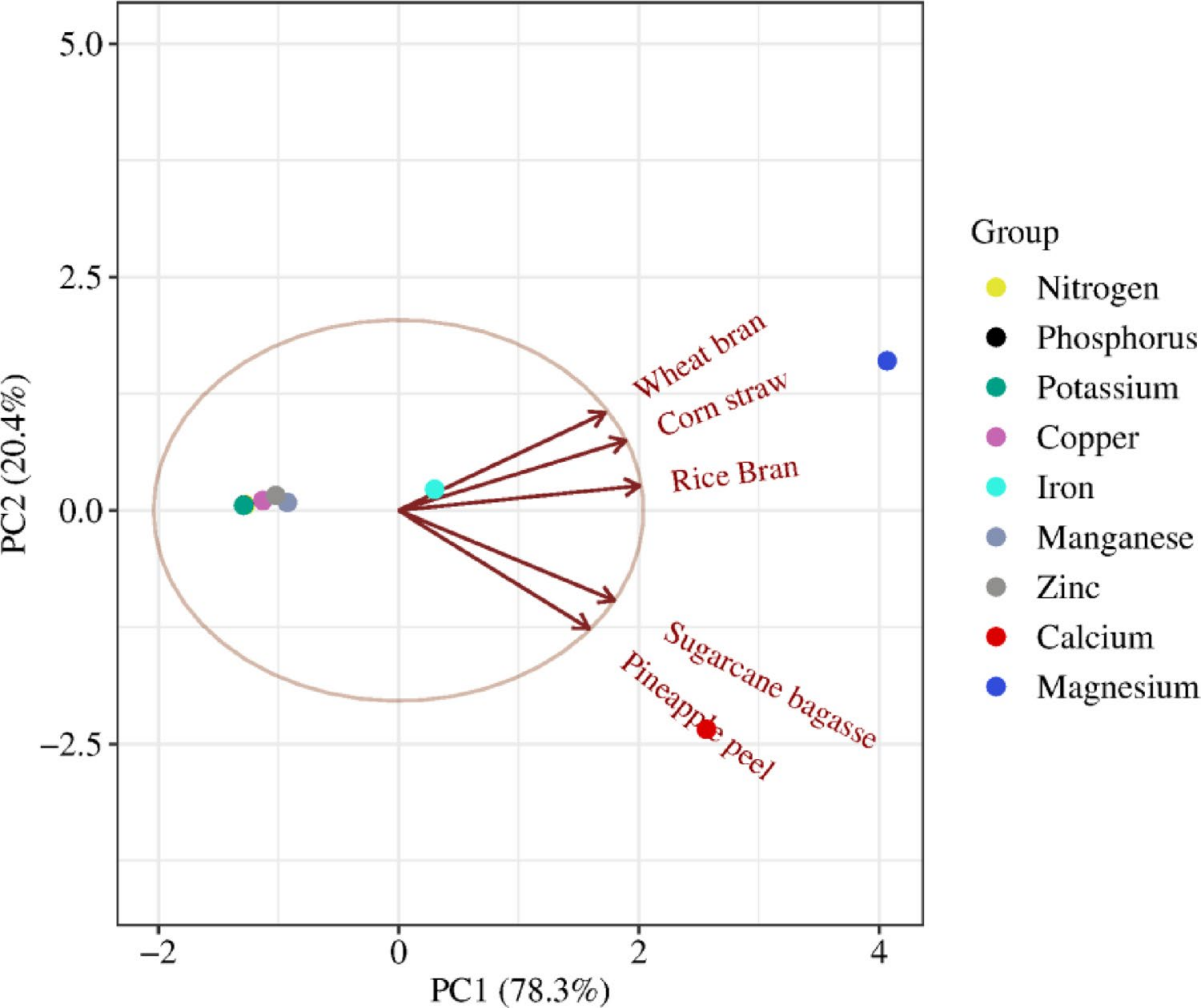
Principal Component Analysis (PCA) biplot of L-ASNase activity in solid-state fermentation (SSF)

The biplot demonstrated clear differences among the chemical compositions of each substrate. The two principal components accounted for 98.7% of the total variance (PC1: 78.3% and PC2: 20.4%). Sugarcane bagasse and pineapple peel exhibited distinct chemical profiles, characterized by lower levels of nutrients such as potassium (K), copper (Cu), and zinc (Zn) (therefore, arrows pointing downwards, isolated from other substrates), which may have contributed to L-asparaginase production. On the other hand, wheat bran, corn straw, and rice flour were associated with higher nitrogen (N) and phosphorus (P) content (Fig. 8). Thus, this analysis allowed for the provided insights into the relationship between the chemical composition of low-cost substrates relates to enzymatic induction, providing hypotheses on which characteristics may be linked to the ability of each substrate to induce L-asparaginase production. The specific values for the chemical analysis of each element studied, the relative humidity, and the apparent density can be observed in supplementary Table 4.

## Discussion

Currently, commercial biopharmaceutical ASNase is of bacterial origin, specifically from *Escherichia coli* and *Erwinia chrysanthemi*. These enzymes have also been investigated for their potential application in modern neoplasms, such as glioblastoma, laryngeal squamous cell carcinoma, and lung adenocarcinoma. However, adverse effects associated with these drugs often preclude long-term administration. The development of hypersensitivity is the primary reason for treatment discontinuation, causing severe allergic reactions in patients (Fontes et al. 2024; Jia et al. 2025).

From the plate screening of the 33 filamentous fungi tested, eight (24%) showed promising results, indicated by the formation of a pink zone on solid media. Enzymatic index (EI) values ranged from 2.67 (corresponding to glutaminase- and urease-free L-asparaginase) to 5.45 for fungi producing L-asparaginase combined with glutaminase. Among the yeasts, one isolate produced glutaminase- and urease-free L-asparaginase, while two others produced it in combination with glutaminase. A similar study using the bromothymol blue (BTB) indicator reported a maximum EI of 7.6 for isolates producing glutaminase- and urease-free L-ASNase, 5.4 for glutaminase-producing but urease-free isolates, and 3.62 for isolates producing both L-ASNase and urease but free of glutaminase after 96 h of incubation. These fungi were also isolated from Antarctic soil and moss (Ashok et al. 2019). The production of L-asparaginase with low glutaminase and urease activity in Antarctica is limited to a few studies involving filamentous fungi (Ashok et al. 2019; Andrade et al. 2023) and yeasts (Freire et al. 2020; Sánchez-Moguel et al. 2023), this highlights the need for further exploration into new fungal sources with therapeutic potential.

In our study, some fungal isolates produced a halo on solid media for L-asparaginase but did not show efficient production in liquid media. While this correlation is not fully elucidated, it is suspected that microbial metabolism is heavily influenced by cultivation conditions (solid vs. liquid) (Cunha et al. 2019), each microorganism has its own genetic makeup specific requirements for enzymatic production. For example, in a solid medium, a fungus can produce a quantity of enzyme that is well expressed, creating a large halo through hyphal growth, but with low enzymatic activity. Consequently, we employed multiple methodologies for a more selective and efficient screening of the isolates (Hatamzadeh et al. 2020). In contrast, submerged fermentation (SmF) utilizes liquid nutrient broth, factors such as hydrogen ion concentration (pH), agitation (rpm), dissolved oxygen (DO) influencing microbial growth, which directly influences enzyme production (Youssef et al. 2025).

In addition to solid media screening, the yeasts were screened using 96-well microplate. Of the 29 yeasts tested, five (17.24%) showed varying levels of coloration. It has been argued that the intensity of the wine/red color in this method is directly proportional to enzymatic activity yields, confirming these yeasts as enzyme producers (Freire et al. 2020). Comparing these results with current literature, a study of 134 Antarctic yeasts using the 96-well microplate method found nine promising ASNase producers (approximately 6.2% of all strains), identified as *Meyerozyma guilliermondii*, *Leucosporidium muscorum*, and *Glaciozyma martinii* (Freire et al. 2020).

The top-performing isolates were further tested in liquid media, where both filamentous fungi and yeasts showed significant color shifts and pH changes, rising from 5.5 to 8.0 in some cases. These results are consistent with the hypothesis that L-ASNase production is accompanied by an increase in culture filtrate pH due to the breakdown of L-asparagine into aspartic acid and ammonia, which alkalinizes the medium. Despite the possibility of false positives, plate assays utilize this principle by incorporating phenol red as a pH indicator, which turns pink under alkaline conditions (Gulati et al. 1997).

Molecular identification of the Antarctic filamentous fungi grouped them with sequences from various genera, including *Pseudogymnoascus*, which has been reported as a producer of agarase, carrageenase, and L-asparaginase (Duarte et al. 2018; Souza et al. 2023). Furthermore, the isolate *Mortierella turficola* FM2.1, an L-asparaginase producer obtained from wood in the South Shetland Archipelago, demonstrated cytotoxic effects on the MIAPaCa-2 cancer cell line. Treatment with crude enzymatic extracts (12.5% to 50%) for 24 h reduced cell viability and caused cytoskeletal and nuclear deformations, suggesting potential applications in pancreatic cancer treatment, especially as no cytotoxic action was observed in non-tumor cells (Flores et al. 2025).

Other genera such as *Cosmospora* sp., *Hypocrea* sp., *Cladosporium* sp., *Penicillium* sp., *Pseudeurotium* sp., *Oidiodendron* sp., and *Acremonium* sp. from diverse terrestrial and marine samples have also been reported as L-asparaginase producers (Duarte et al. 2018). Since only a few Antarctic yeast species like *Leucosporidium muscorum*, *L. scottii*, and *Glaciozyma martinii* predominate in the literature (Freire et al. 2020; Sánchez-Moguel et al. 2023) the isolates described in this work may represent additional producers of this enzyme.

The Antarctic yeast *Leucosporidium muscorum* CRM 1648, isolated from marine sediment showed initial activity in the quantitative analysis of ASNase production (490.41 U/L), as the highest producer. However, through optimization, ASNase production became more evident. The researchers observed that the highest amounts of the enzyme were produced when sucrose was used as a carbon source (1028.3 ± 81.4 U/L), followed by sorbitol (895.8 ± 77.5 U/L) and glucose (878.8 ± 32.8 U/L). However, glycerol and xylose had a smaller impact on ASNase production (439.6 ± 28.9 U/L and 471.7 ± 59.7 U/L, respectively) (Freire et al. 2020).

In a study conducted on King George Island, fungi isolated fungi isolated from plant specimens *Polytrichastrum alpinum* and *Sanionia uncinata* (strains *Collariella* sp. MSA10, *Peroneutypa* sp. MSA13, and *Epicoccum* sp. MPA01) showed L-ASNase production between 1.13 and 1.29 U/g. In contrast, their glutaminase and urease activities were relatively low, typically not exceeding 0.66 U/g (Andrade et al. 2023),

In this study, solid-state fermentation (SSF) was utilized as a sustainable and cost-effective alternative for enzyme production. Our findings demonstrate the versatility of Antarctic *V. victoriae* in utilizing different substrates, contributing to more sustainable bioprocessing. While filamentous fungi are common enzyme producers, yeasts are increasingly being studied in SSF. For instance, *V. victoriae* L92 and *Leucosporidium scottii* L120, isolated from Antarctic lichens and deep-sea sediments, produced 0.9 and 1.1 U/g of L-ASNase, respectively, using an inert polyurethane support (Correa et al. 2020). Our PCA showed that substrate nutrients influence L-ASNase production. Substrates with lower levels of metal ions such as K, Cu, and Zn tended to positively influence enzymatic production, which may help explain the high yield observed with pineapple peel that it had a statistically different L-ASNase production compared to the other substrates tested (Lincoln et al. 2015; Cunha et al. 2019).

The average L-ASNase production values, particularly for the yeast *V. victoriae* G.L11, represent a finding with limited correspondence in the existing literature. While some fungi produced glutaminase- and urease-free L-ASNase, others produced it alongside glutaminase (Ratuchne et al. 2023). To the best of our knowledge, studies involving agro-industrial by-products for SSF by Antarctic yeasts to produce L-asparaginase appear to be unknown, making these findings highly attractive for future research.

## Conclusion

The present study has elucidated critical aspects of the selection and production of L-asparaginase by fungi from diverse Antarctic genera, such as *Antarctomyces*. The screening process for L-asparaginase-producing fungi on solid media proved to be efficient, allowing for a clear correlation between the formation of halos on solid Czapek Dox medium and the production of extracellular L-asparaginase.

According to the results, the yeast strains specifically the isolate *V. victoriae* G.L11 demonstrated the most significant results, producing L-asparaginase with low glutaminase and urease activities compared to the other isolates. The production of L-asparagine using a low-cost substrate was promising, especially with pineapple peel waste, as it could reduce future enzyme production costs. However, it is important to emphasize that downstream processes are generally considered the most expensive phase of biomanufacturing, and enzymes produced in complex, nutrient-rich media—such as agroindustrial waste substrates—require a well-planned purification workflow. These findings underscore the need for future optimization and purification of the enzyme produced by this strain, which may possess unique characteristics that are highly desirable for pharmaceutical or biotechnological applications.

## Supplementary Information

Below is the link to the electronic supplementary material.

Supplementary Material 1

## Data Availability

No datasets were generated or analysed during the current study.
