# Supplementary material for: L-asparaginase by filamentous fungi and yeasts from lichens and soils across maritime Antarctic islands
Source: Extremophiles. 2026 Aug 1;30(1):25. doi: 10.1007/s00792-026-01437-2 (PMC13428764; doi:10.1007/s00792-026-01437-2)
Supplement: Supplementary file 1 — Supplementary Material 1 [file 792_2026_1437_MOESM1_ESM.docx]

**Supplementary Figure 1.** Obtaining soil samples from different locations in the South Shetland Archipelago (A). Processing samples to obtain isolates (B).

.
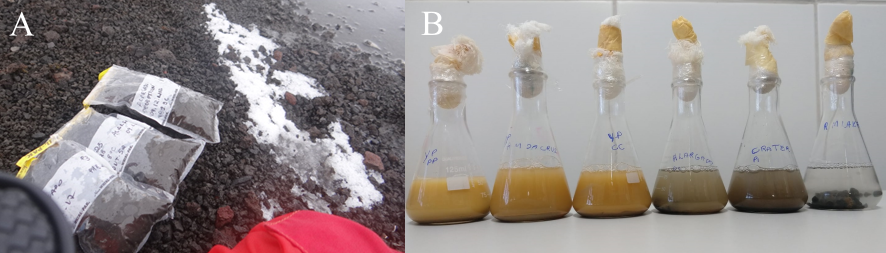


**Supplementary Figure 2.** Linear range of the AHA standard curve, the regression equation and R-squared value.

**Supplementary Figure 3.** Flowchart of the total number of isolates screened and quantified in liquid medium and solid-state fermentation.


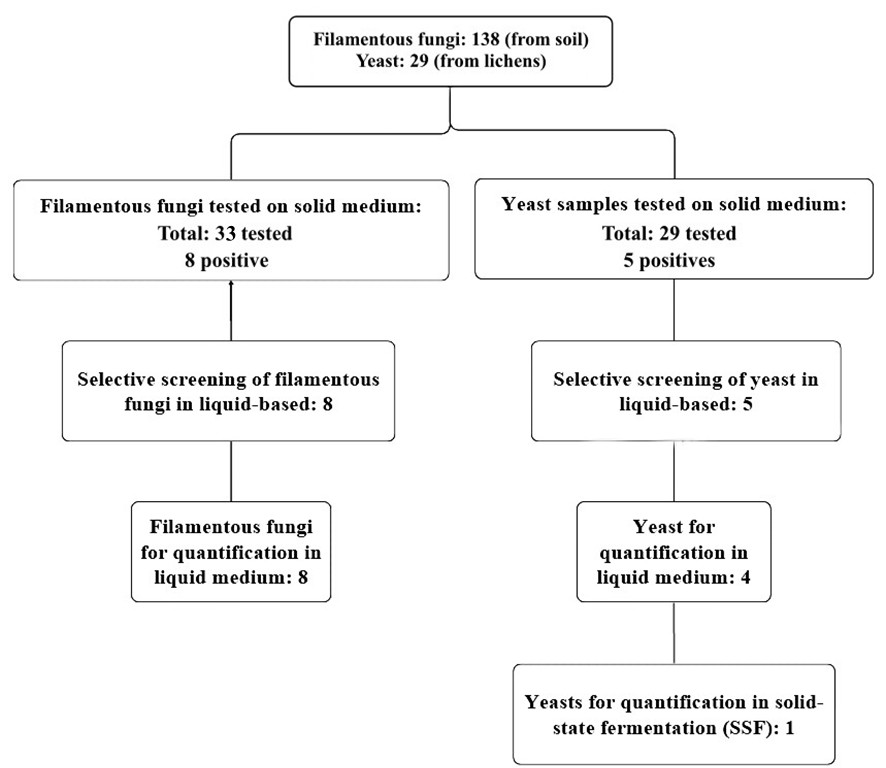


**Supplementary Fig. 4.** Screening of L-ASNase by filamentous fungi from Antarctica in different nitrogen sources.

| **Isolated** | **Nitrate** | **Asparagine** | **Glutamine** | **Urea** |
| --- | --- | --- | --- | --- |
| F1.CR.LASP | **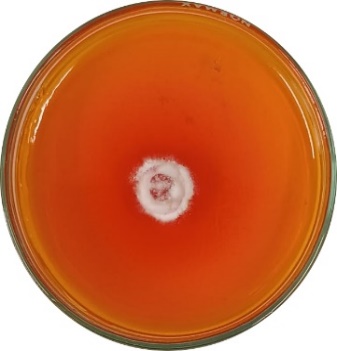** | **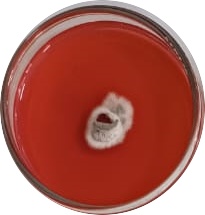** | **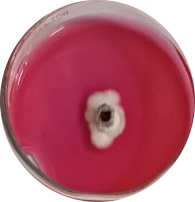** | **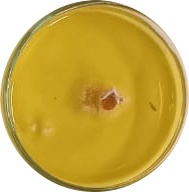** |
| F2.CR.LASP | 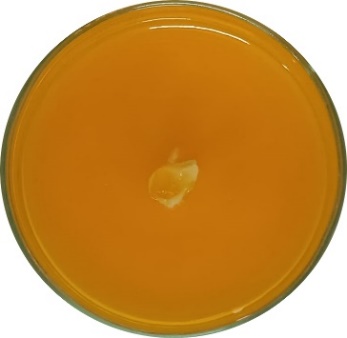 | 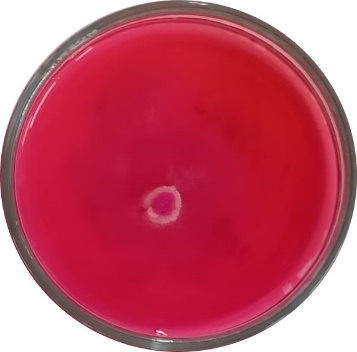 | 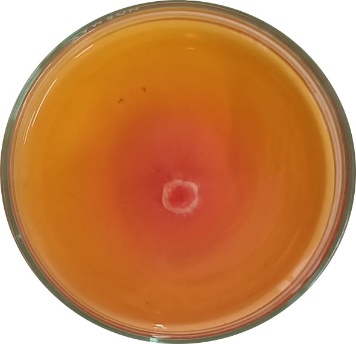 | 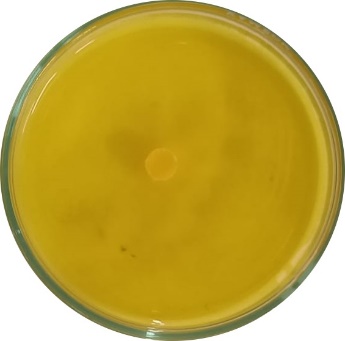 |
| F3.CR.LASP | 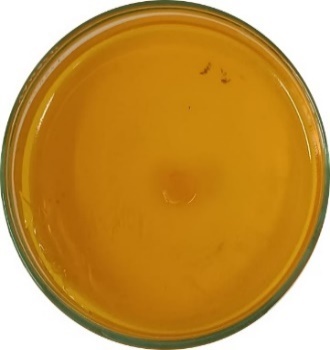 | 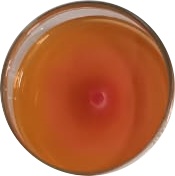 | 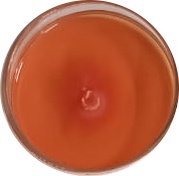 | 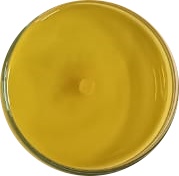 |
| F4.CR.LASP | 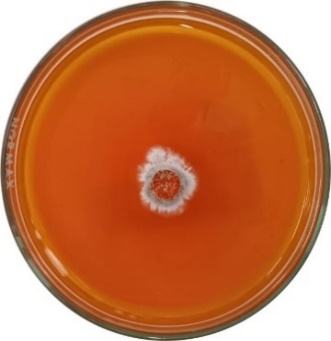 | 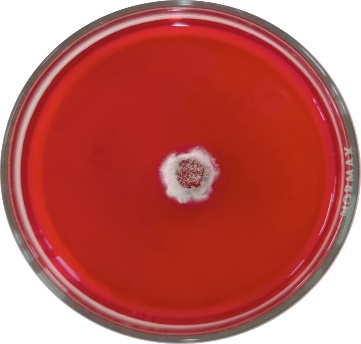 | 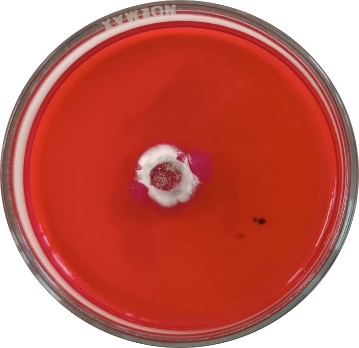 | 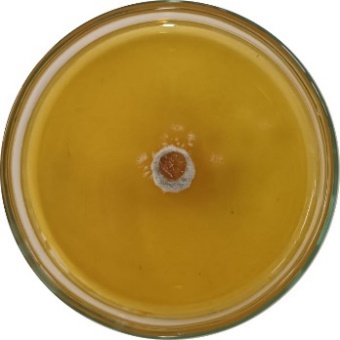 |
| F6.CR.LASP | 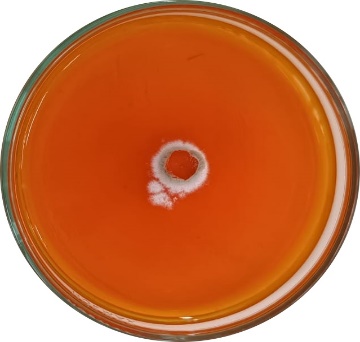 | 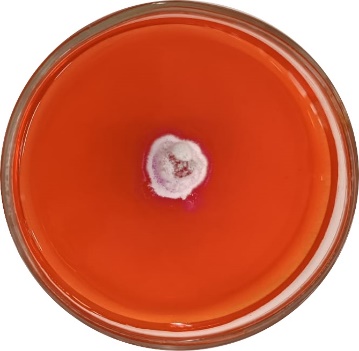 | 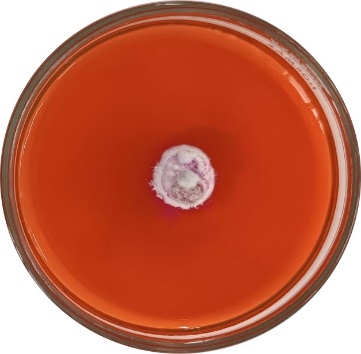 | 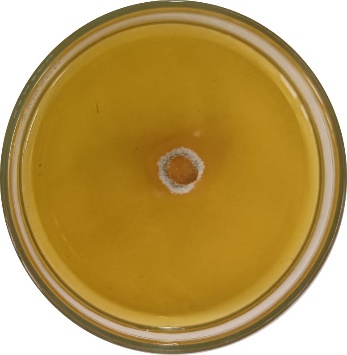 |
| F7.CR.LASP | 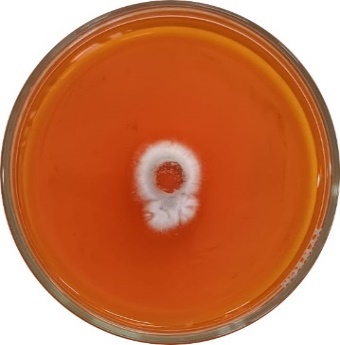 | 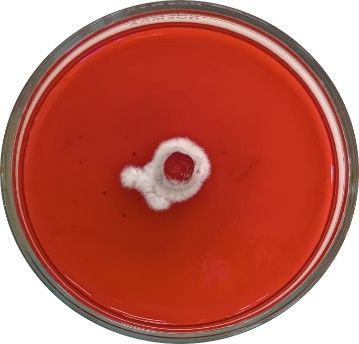 | 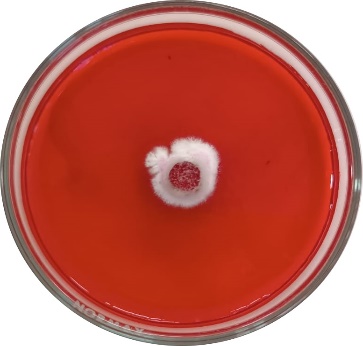 | 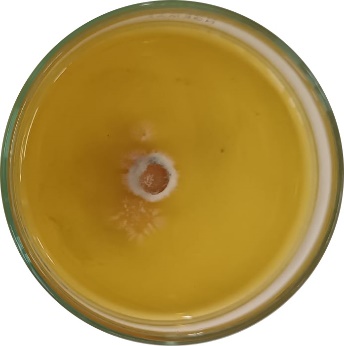 |
| F9.CR.LASP | 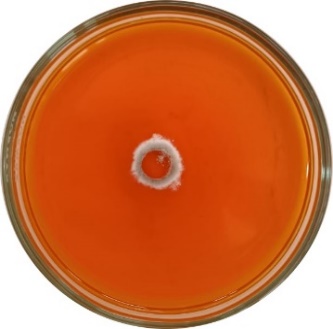 | 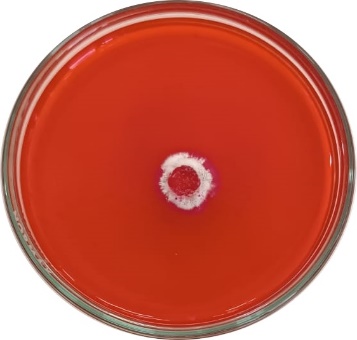 | 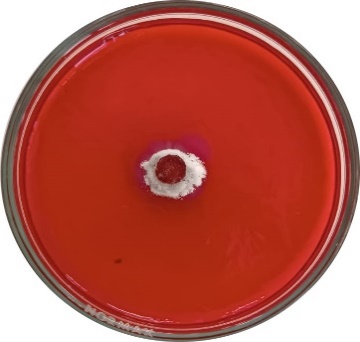 | 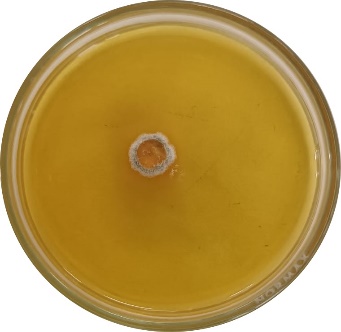 |
| F8.CR.LASP | 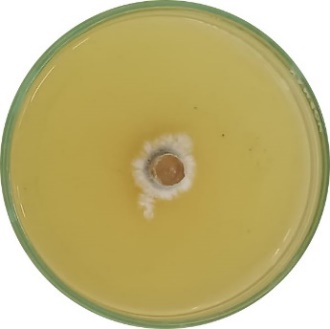 | 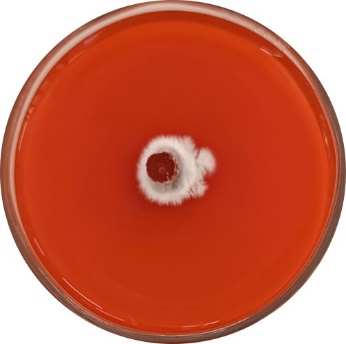 | 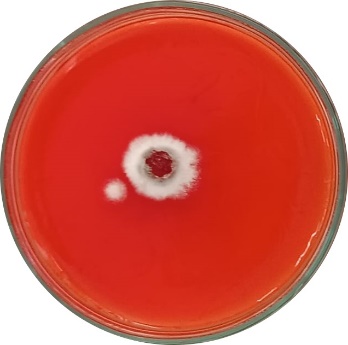 | 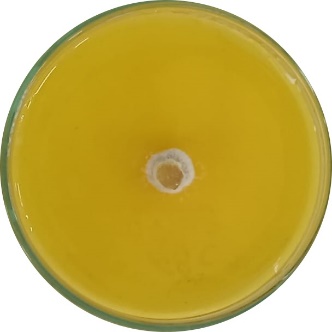 |
| F11.CR.LASP | 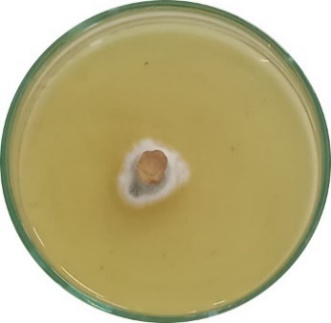 | 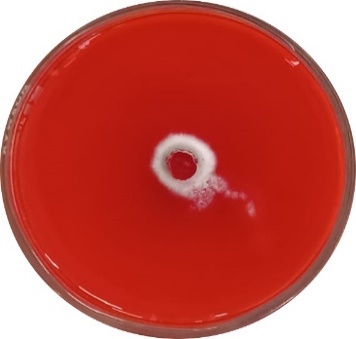 | 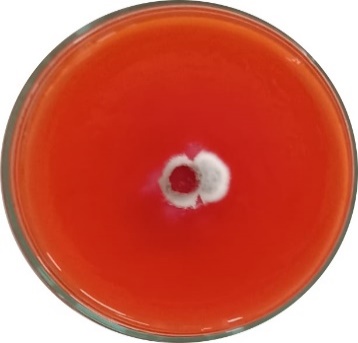 | 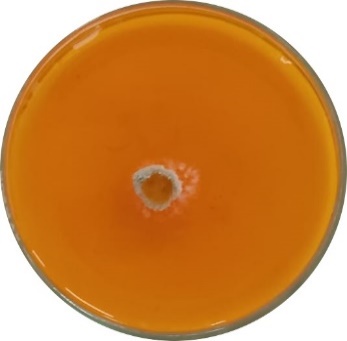 |
| F12.CR.LASP | 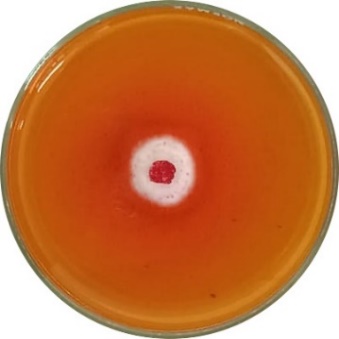 | 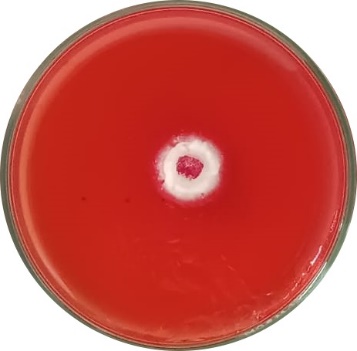 | 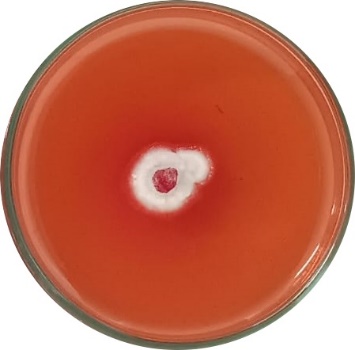 | 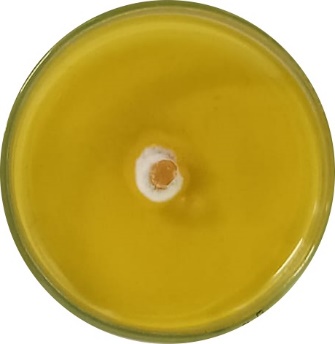 |
| *Antarctomyces* sp. F13.CR.LASP | 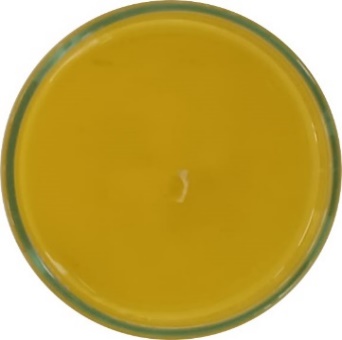 | 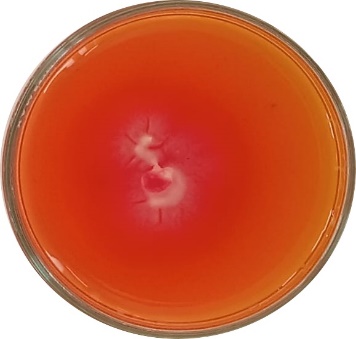 | 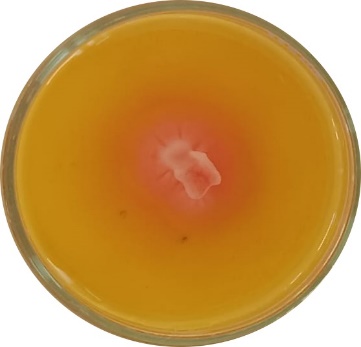 | 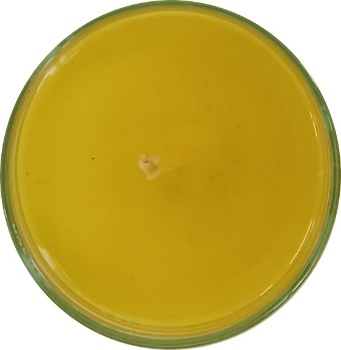 |
| F14.CR.LASP | 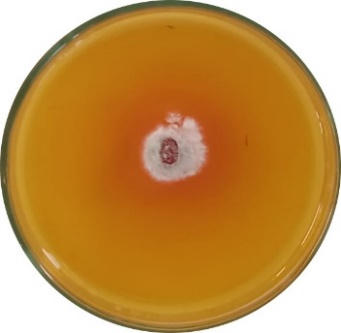 | 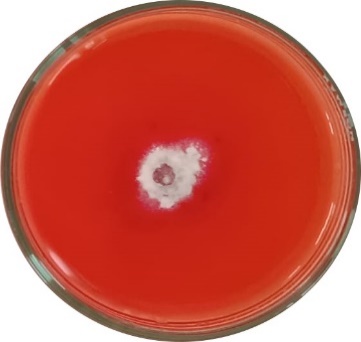 | 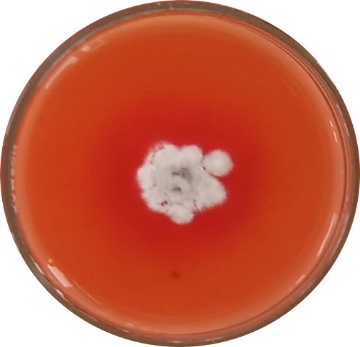 | 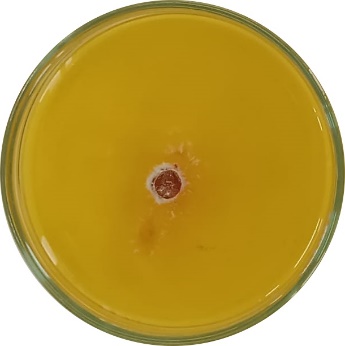 |
| F1.CC.LASP | 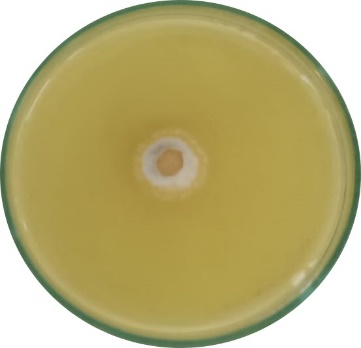 | 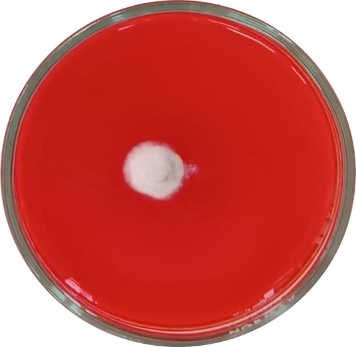 | 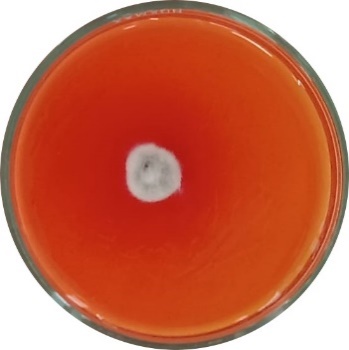 | 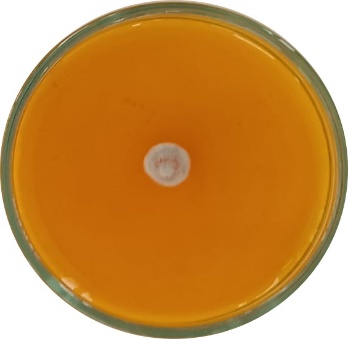 |
| F2.CC.LASP | 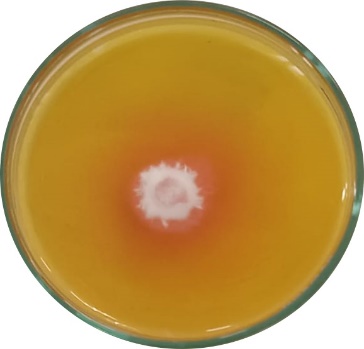 | 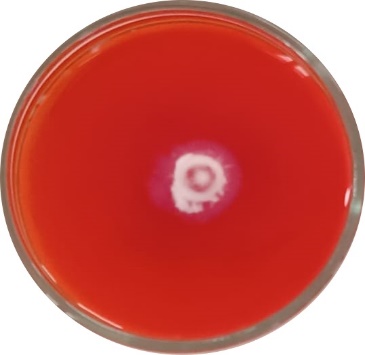 | 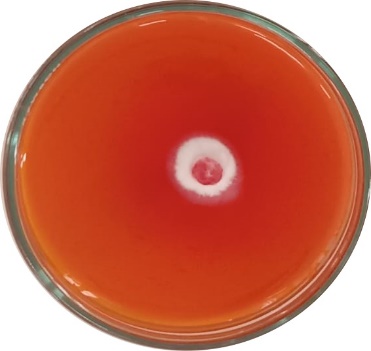 | 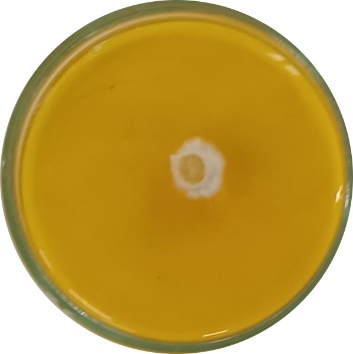 |
| F4.CC.LASP | 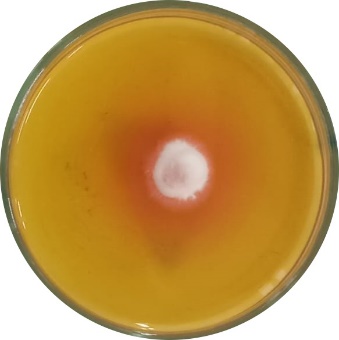 | 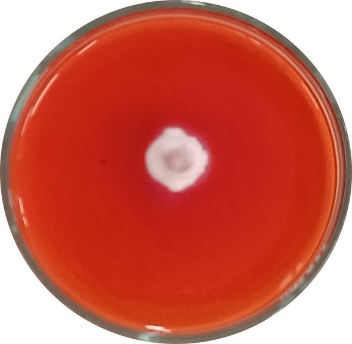 | 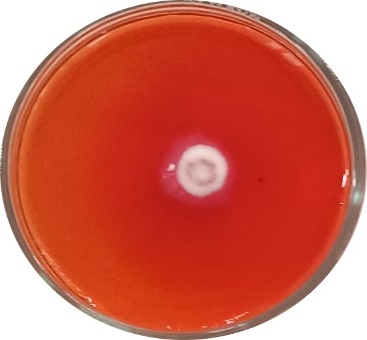 | 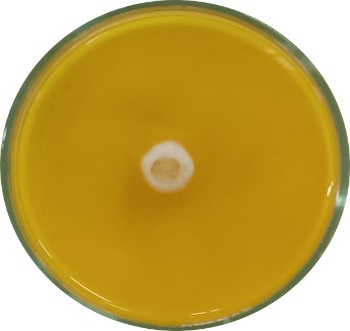 |
| *Pseudogymnoascus* sp. F6.CC.LASP | 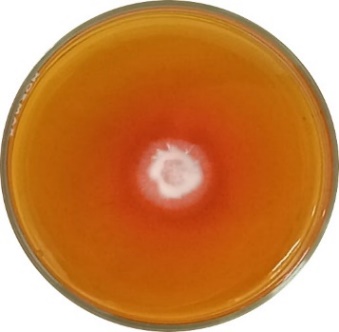 | 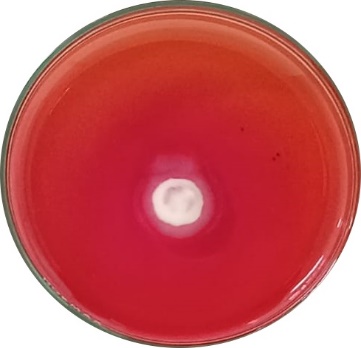 | 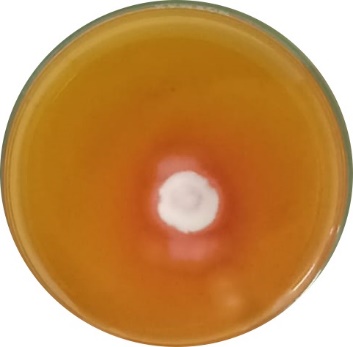 | 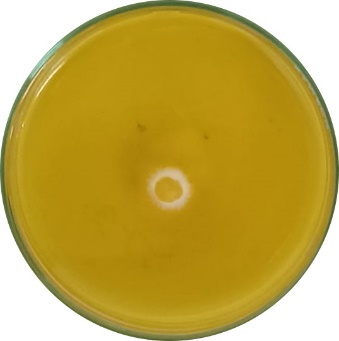 |
| F7.CC.LASP | 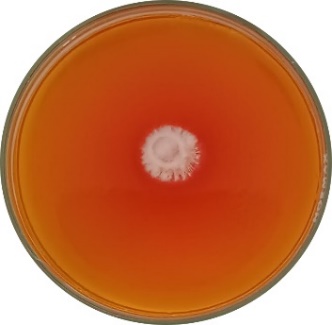 | 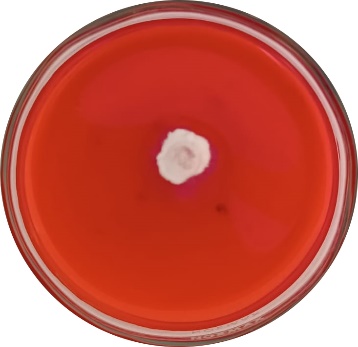 | 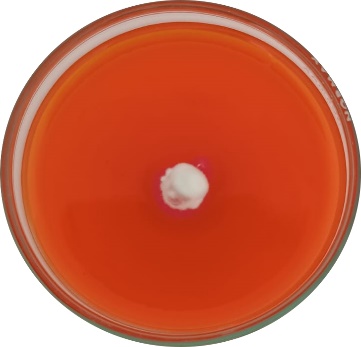 | 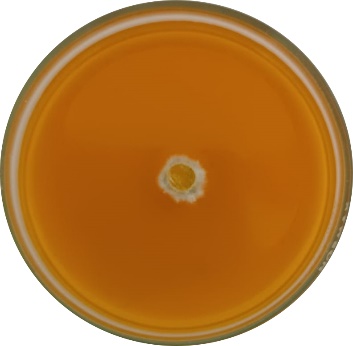 |
| F8.CC.LASP | 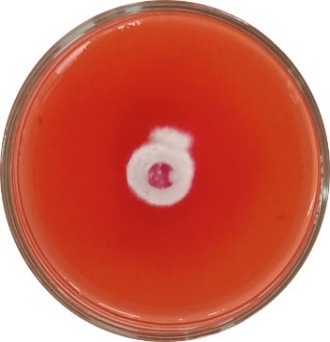 | 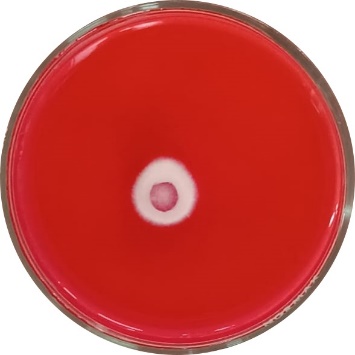 | 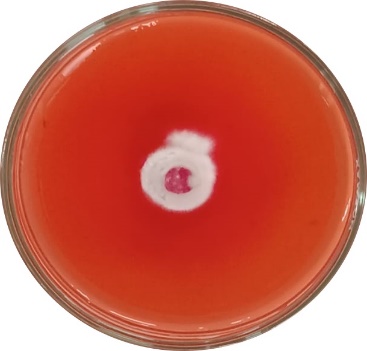 | 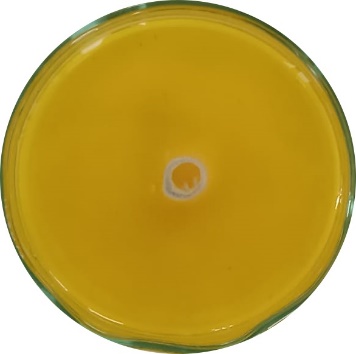 |
| F1.AL.LASP | 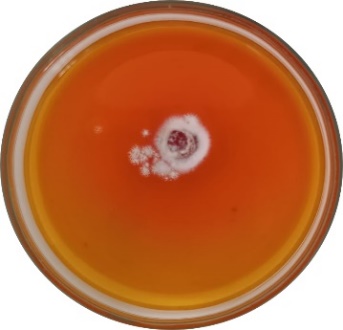 | 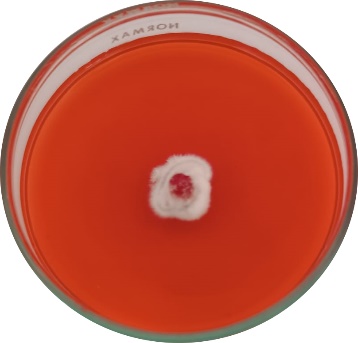 | 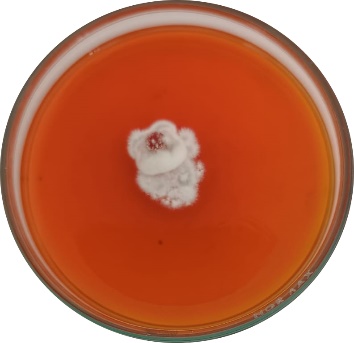 | 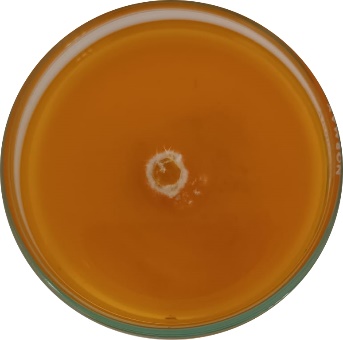 |
| F1.PP.LASP | 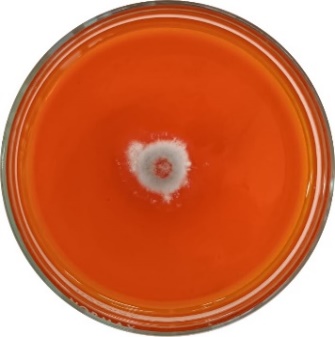 | 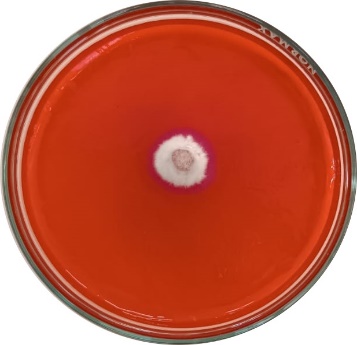 | 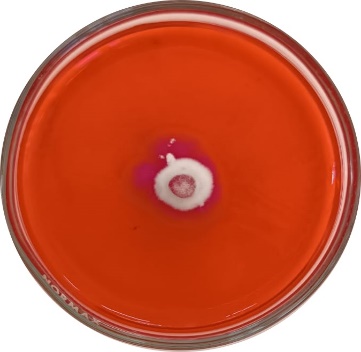 | 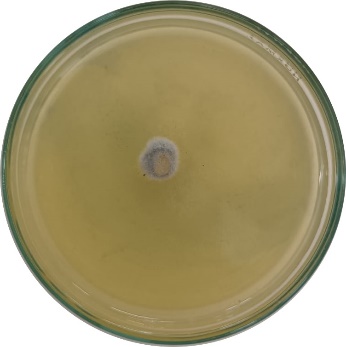 |
| F2.PP.LASP | 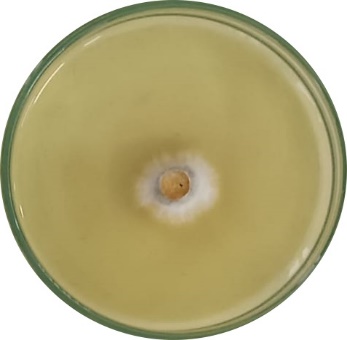 | 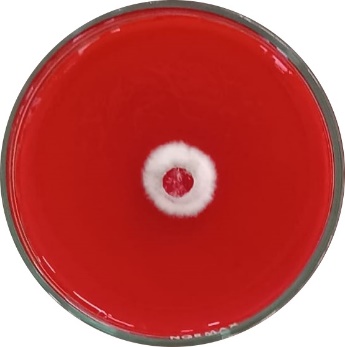 | 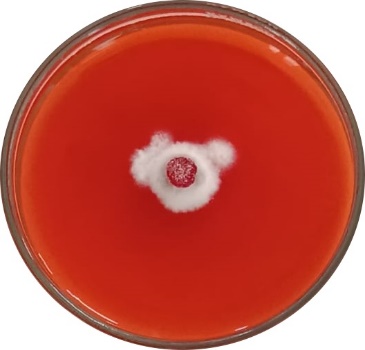 | 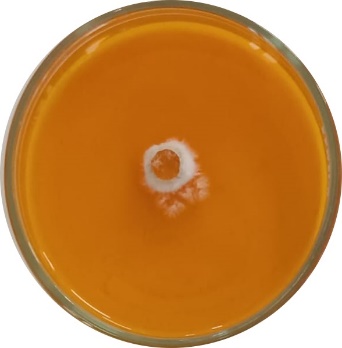 |
| F3.PP.LASP | 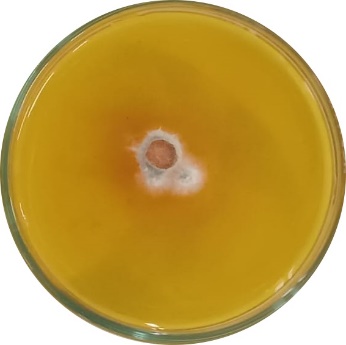 | 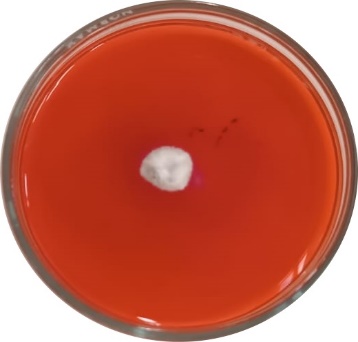 | 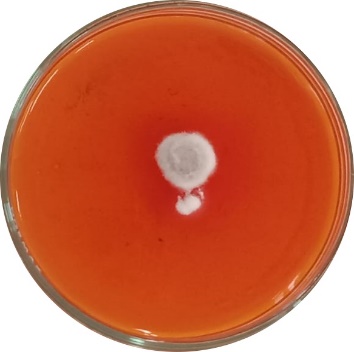 | 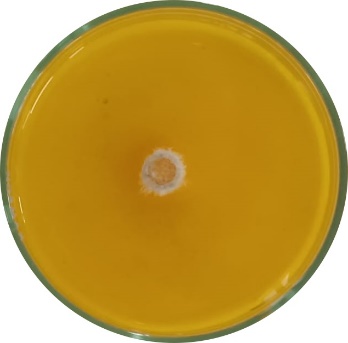 |
| F1.ADC.LASP | 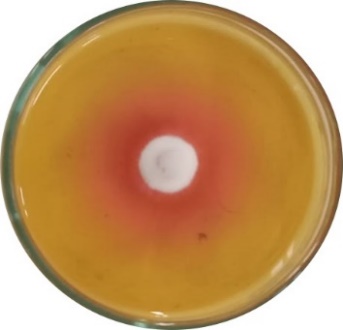 | 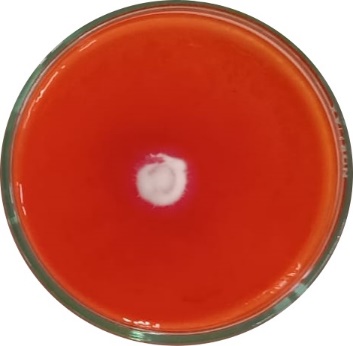 | 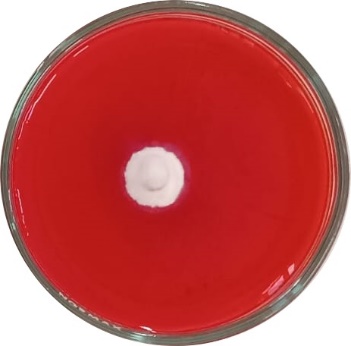 | 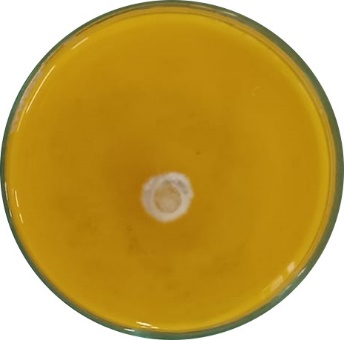 |
| F2.ADC.LASP | 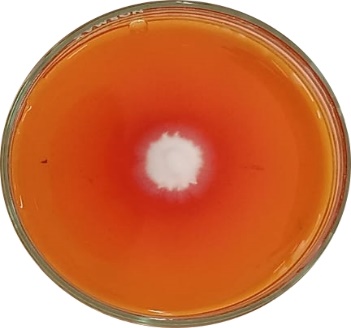 | 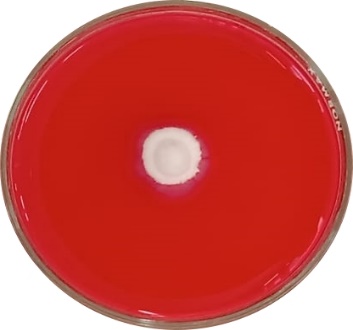 | 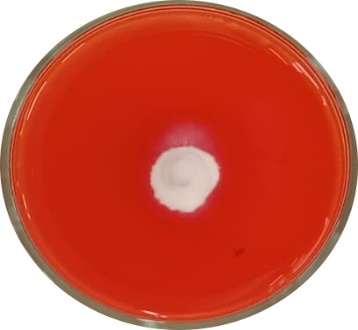 | 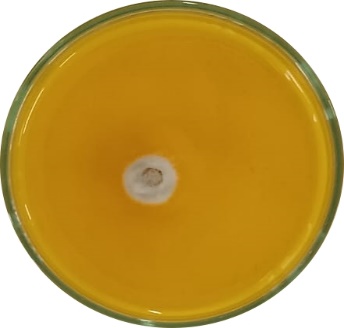 |
| F1.FBPI.LASP | 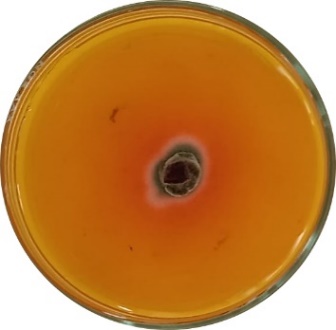 | 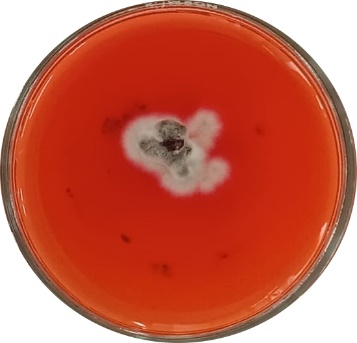 |  |  |
| F1.FBP2.LASP |  |  |  |  |
| *Cladosporium* sp. F2.FBP2.LASP |  |  |  |  |

**Supplementary Table 1.** Quantification and total proteins of L-asparagine, glutaminase, and urease by filamentous fungi from Antarctica.

|  | **L-asparaginase** |  | **Glutaminase** |  | **Urease** |  |  |
| --- | --- | --- | --- | --- | --- | --- | --- |
| **Fungal isolates** | **Enzymatic Activity (U/L)** | **Specific activity (U/g)** | **Enzymatic Activity (U/L)** | **Specific activity (U/ug)** | **Enzymatic Activity (U/L)** | **Specific activity (U/g)** | **Total protein (ug/mL)** |
| F4.PP.LASP | 0.11 | 0.0004 | 0.14 | 0.0006 | 0.17 | 0.0007 | 242.89 |
| F5.CC.LASP | 0.15 | 0.0005 | 0.14 | 0.0005 | 0.20 | 0.0007 | 276.22 |
| F5.CR.LASP | 0.05 | 0.0003 | 0.22 | 0.0014 | 0.14 | 0.0009 | 151.78 |
| F13.CR.LASP | 0.76 | 0.0043 | 0.82 | 0.0046 | 0.84 | 0.0048 | 176.22 |
| F2.FBP2.LASP | 0.15 | 0.0009 | 0.26 | 0.0015 | 0.20 | 0.0012 | 172.22 |
| F15.CR.LASP | 0.06 | 0.0003 | 0.20 | 0.0011 | 0.24 | 0.0014 | 175.11 |
| F6.CC.LASP | - | - | 0.05 | 0.0003 | 0.24 | 0.0014 | 165.00 |
| F2.CR.LASP | - | - | 0.04 | 0.0002 | 0.28 | 0.0014 | 204,00 |

**Supplementary Table 2.** Quantification and total proteins of L-asparagine, glutaminase, and urease by filamentous yeast from Antarctica.

|  | **L-asparaginase** |  | **Glutaminase** |  | **Urease** |  |  |
| --- | --- | --- | --- | --- | --- | --- | --- |
| **Isoladed** | **Enzymatic Activity (U/L)** | **Specific activity (U/g)** | **Enzymatic Activity (U/L)** | **Specific activity (U/g)** | **Enzymatic Activity (U/L)** | **Specific activity (U/g)** | **Total protein (ug/mL)** |
| 7.L25 | 0.03 | 0.0002 | 0.12 | 0.0007 | - | - | 176.22 |
| K.L24 | 0.09 | 0.0003 | 0.21 | 0.0007 | 0.005 | - | 341.78 |
| G.L11 (*Vishniacozyma victorie*) | 0.17 | 0.0006 | 0.11 | 0.0004 | 0.06 | 0.0002 | 281.78 |
| 4.L1 (*Vishniacozyma victorie*) | 0.74 | 0.0030 | 0.77 | 0.0031 | 0.75 | 0.0030 | 246.22 |

**Supplementary Table 3.** Summary of the Analysis of Variance (ANOVA) applied to low-cost substrates produced by Antarctic yeast (*Vishniacozyma victoriae* G.L11)

| **Sources of variation (SV)** | **Degress of freedom (GL)** | **Sum of squares (SQ)** | **Mean square (QM)** | **F calculated (Fc)** | **P value** |
| --- | --- | --- | --- | --- | --- |
| Substrates | 4 | 0.695180 | 0.173795 | 18.221 | P<0.0000 |
| Erro | 40 | 0.381536 | 0.009538 |  |  |
|  |  |  |  |  |  |
|  |  |  |  |  |  |
| **Substrate** | **Mean** |  |  |  |  |
| Pineapple peel | 0.5171 |  |  |  |  |
| Sugarcane bagasse | 0.3396 |  |  |  |  |
| Corn straw | 0.3798 |  |  |  |  |
| Wheat bran | 0.4048 |  |  |  |  |
| Rice bran | 0.1367 |  |  |  |  |

**Supplementary Table 4**. Chemical composition of each low-cost substrate used in solid-state fermentation for L-asparaginase production.

|  | **Low-cost substrate** | | | | |
| --- | --- | --- | --- | --- | --- |
| **Chemical component** | **Wheat bran** | **Pineapple peel** | **Sugarcane Bagasse** | **Corn straw** | **Rice flour** |
| Total Nitrogen (%) | 2.52 | 0.81 | 0.33 | 0.56 | 1.49 |
| Phosphorus - P2O5 (%) | 2.00 | 0.30 | 0.20 | 0.49 | 0.23 |
| Potassium - K2O (%) | 0.79 | 1.19 | 0.65 | 0.49 | 0.06 |
| Copper (mg/kg) | 32.7 | 6.99 | 1.29 | 3.03 | 12.0 |
| Iron (mg/kg) | 179.0 | 99.6 | 203.0 | 215.0 | 55.2 |
| Manganese (mg/kg) | 162.0 | 85.4 | 18.3 | 14.2 | 11.3 |
| Zinc (mg/kg) | 79.10 | 4.70 | 11.2 | 16.0 | 15.1 |
| Calcium (mg/kg) | 257.0 | 1266.0 | 782.0 | 193.0 | 78.9 |
| Magnesium (mg/kg) | 1966.0 | 504.0 | 452.0 | 646.0 | 135.0 |
| Percentage of humidity (%) | 1.04 | 0.71 | 0.47 | 0.68 | 1.02 |
| Apparent density (mL) | 14.08 | 8.75 | 40.0 | 13.0 | 7.0 |
